# Supplementary material for: A new large hypercarnivorous crocodyliform from the Maastrichtian of Southern Patagonia, Argentina
Source: PLoS One. 2025 Aug 27;20(8):e0328561. doi: 10.1371/journal.pone.0328561 (PMC12385380; doi:10.1371/journal.pone.0328561)
Supplement: Attachment — Submitted filename: Review - PONE-D-25-04716.pdf [file pone.0328561.s003.pdf]

PLOS ONE

A new large hypercarnivorous crocodyliform from theMaastrichtian of Southern Patagonia, Argentina

--Manuscript Draft--

**Manuscript Number:**	PONE-D-25-04716
**Article Type:**	Research Article
**Full Title:**	A new large hypercarnivorous crocodyliform from theMaastrichtian of Southern Patagonia, Argentina
**Short Title:**	New large peirosaurid from Patagonia
**Corresponding Author:**	Diego Pol<br>CONICET: Consejo Nacional de Investigaciones Cientificas y Tecnicas<br>Buenos Aires, ARGENTINA
**Keywords:**	Crocodyliformes, Peirosauridae, Chorrillo Formation, Patagonia, Maastrichtian, Late Cretaceous
**Abstract:**	The first crocodyliform specimen from the Maastrichtian Chorrillo Formation (Austral Basin, Patagonia) is here described. The discovery was made about 30 km to the SW of the town of El Calafate (Province of Santa Cruz, Argentina) and consists of a beautifully preserved and articulated skull and jaws, and part of the postcranial skeleton that was preserved encased in a large concretion. This new taxon belongs to the notosuchian clade Peirosauridae, representing the latest and southernmost record for this group of crocodyliforms. The new taxon is recovered as closely related to other robust and broad-snouted peirosaurids that lived by the end of the Cretaceous Period, such as Colhuehuapisuchus from the Maastrichtian of Central Patagonia and Miadanasuchus oblita from the Maastrichtian of Madagascar. The completeness of the new specimenreveals, for the first time, the anatomy and body plan of a large and broad snouted peirosaurid. The new taxon bears large ziphodont teeth, a broad oreinirostral snout that is only slightly longer than 50% the skull length, and a deep adductor chamber in the temporal region and posterior mandibular ramus. CT scan study reveals a nasal cavity flanked by a large, but anteriorly restricted, antorbital sinus within the maxilla. The new crocodyliform completes the predatorial component of terrestrial ecosystems at high paleolatitudes by the end of the Cretaceous Period.
**Order of Authors:**	Fernando Novas
	Diego Pol
	Federico Agnolin
	Ismar Souza Carvalho
	Makoto Manabe
	Takanobu Tsujishi
	Sebastián Rozadilla
	Gabriel Lio
	Marcelo Isasi
**Additional Information:**	
**Question**	**Response**
**Financial Disclosure**<br><br>Enter a financial disclosure statement that describes the sources of funding for the work included in this submission. Review the submission guidelines for detailed requirements. View published research	Yes

Powered by Editorial Manager® and ProduXion Manager® from Aries Systems Corporation

articles from *PLOS ONE* for specific examples.<br><br>This statement is required for submission and **will appear in the published article** if the submission is accepted. Please make sure it is accurate.<br><br>**Funded studies**<br>Enter a statement with the following details:<br>• Initials of the authors who received each award<br>• Grant numbers awarded to each author<br>• The full name of each funder<br>• URL of each funder website<br>• Did the sponsors or funders play any role in the study design, data collection and analysis, decision to publish, or preparation of the manuscript?<br><br>Did you receive funding for this work?	
Please add funding details.<br>as follow-up to "**Financial Disclosure**<br><br>Enter a financial disclosure statement that describes the sources of funding for the work included in this submission. Review the submission guidelines for detailed requirements. View published research articles from *PLOS ONE* for specific examples.<br><br>This statement is required for submission and **will appear in the published article** if the submission is accepted. Please make sure it is accurate.	DP<br>9282-R-22<br>National Geographic Society<br>https://www.nationalgeographic.org/society/<br>The funders had no role in study design, data collection and analysis, decision to publish, or preparation of the manuscript.<br><br>ISC<br>Faperj E-26/200.998/2024<br>Fundação Carlos Chagas Filho de Amparo à Pesquisa do Estado do Rio de Janeiro<br>https://www.faperj.br/<br>The funders had no role in study design, data collection and analysis, decision to publish, or preparation of the manuscript.<br><br>ISC<br>CNPq 303596/2016-3<br>Conselho Nacional de Desenvolvimento Científico e Tecnológico<br>https://www.gov.br/cnpq/pt-br<br>The funders had no role in study design, data collection and analysis, decision to publish, or preparation of the manuscript.

Powered by Editorial Manager® and ProduXion Manager® from Aries Systems Corporation

**Funded studies**<br>Enter a statement with the following details:<br>• Initials of the authors who received each award<br>• Grant numbers awarded to each author<br>• The full name of each funder<br>• URL of each funder website<br>• Did the sponsors or funders play any role in the study design, data collection and analysis, decision to publish, or preparation of the manuscript?<br><br>Did you receive funding for this work?"	
Please select the country of your main research funder (please select carefully as in some cases this is used in fee calculation).<br>as follow-up to "**Financial Disclosure**<br><br>Enter a financial disclosure statement that describes the sources of funding for the work included in this submission. Review the submission guidelines for detailed requirements. View published research articles from *PLOS ONE* for specific examples.<br><br>This statement is required for submission and **will appear in the published article** if the submission is accepted. Please make sure it is accurate.<br><br>**Funded studies**<br>Enter a statement with the following details:<br>• Initials of the authors who received each award<br>• Grant numbers awarded to each author<br>• The full name of each funder<br>• URL of each funder website<br>• Did the sponsors or funders play any role in the study design, data collection and analysis, decision to publish, or preparation of the manuscript?<br><br>Did you receive funding for this work?"	ARGENTINA - AR
**Competing Interests**	The authors have declared that no competing interests exist.

Use the instructions below to enter a competing interest statement for this submission. On behalf of all authors, disclose any competing interests that could be perceived to bias this work—acknowledging all financial support and any other relevant financial or non-financial competing interests.<br><br>This statement is required for submission and **will appear in the published article** if the submission is accepted. Please make sure it is accurate and that any funding sources listed in your Funding Information later in the submission form are also declared in your Financial Disclosure statement.<br><br>View published research articles from *PLOS ONE* for specific examples.<br><br>**NO authors have competing interests**<br><br>Enter: *The authors have declared that no competing interests exist.*<br><br>**Authors with competing interests**<br><br>Enter competing interest details beginning with this statement:<br><br>*I have read the journal's policy and the authors of this manuscript have the following competing interests: [insert competing interests here]*<br><br>* typeset	
**Ethics Statement**<br><br>Enter an ethics statement for this submission. This statement is required if the study involved:<br><br>• Human participants<br>• Human specimens or tissue<br>• Vertebrate animals or cephalopods<br>• Vertebrate embryos or tissues	Secretaría de Cultura de la Provincia de Santa Cruz

Powered by Editorial Manager® and ProduXion Manager® from Aries Systems Corporation

• Field research<br><br>Write "N/A" if the submission does not require an ethics statement.<br><br>General guidance is provided below. Consult the submission guidelines for detailed instructions. **Make sure that all information entered here is included in the Methods section of the manuscript.**	

**Format for specific study types**<br><br>**Human Subject Research (involving human participants and/or tissue)**<br>• Give the name of the institutional review board or ethics committee that approved the study<br>• Include the approval number and/or a statement indicating approval of this research<br>• Indicate the form of consent obtained (written/oral) or the reason that consent was not obtained (e.g. the data were analyzed anonymously)<br><br>**Animal Research (involving vertebrate animals, embryos or tissues)**<br>• Provide the name of the Institutional Animal Care and Use Committee (IACUC) or other relevant ethics board that reviewed the study protocol, and indicate whether they approved this research or granted a formal waiver of ethical approval<br>• Include an approval number if one was obtained<br>• If the study involved *non-human primates*, add *additional details* about animal welfare and steps taken to ameliorate suffering<br>• If anesthesia, euthanasia, or any kind of animal sacrifice is part of the study, include briefly which substances and/or methods were applied<br><br>**Field Research**<br><br>Include the following details if this study involves the collection of plant, animal, or other materials from a natural setting:<br>• Field permit number<br>• Name of the institution or relevant body that granted permission	
**Data Availability**<br><br>Authors are required to make all data underlying the findings described fully available, without restriction, and from the time of publication. PLOS allows rare exceptions to address legal and ethical concerns. See the PLOS Data Policy and FAQ for detailed information.	Yes - all data are fully available without restriction

A Data Availability Statement describing where the data can be found is required at submission. Your answers to this question constitute the Data Availability Statement and **will be published in the article**, if accepted.<br><br>**Important:** Stating 'data available on request from the author' is not sufficient. If your data are only available upon request, select 'No' for the first question and explain your exceptional situation in the text box.<br><br>Do the authors confirm that all data underlying the findings described in their manuscript are fully available without restriction?	
**Describe where the data may be found in full sentences. If you are copying our sample text, replace any instances of XXX with the appropriate details.**<br><br>• If the data are **held or will be held in a public repository**, include URLs, accession numbers or DOIs. If this information will only be available after acceptance, indicate this by ticking the box below. For example: *All XXX files are available from the XXX database (accession number(s) XXX, XXX.*).<br>• If the data are all contained **within the manuscript and/or Supporting Information files**, enter the following: *All relevant data are within the manuscript and its Supporting Information files.*<br>• If neither of these applies but you are able to provide **details of access elsewhere**, with or without limitations, please do so. For example:<br><br>*Data cannot be shared publicly because of [XXX]. Data are available from the XXX Institutional Data Access / Ethics Committee (contact via XXX) for researchers who meet the criteria for access to confidential data.*<br><br>*The data underlying the results presented in the study are available from (include the name of the third party*	All relevant data are within the manuscript and its Supporting Information files.

and contact information or URL).<br>• This text is appropriate if the data are owned by a third party and authors do not have permission to share the data.<br><br>* typeset	
Additional data availability information:	

Manuscript Click here to access/download;Manuscript;Novas et al Kostensuchus ms.submitted.doc

A new large hypercarnivorous crocodyliform from the Maastrichtian of Southern Patagonia, Argentina

Fernando E. Novas[1,2,3], Diego Pol[2,4*], Federico L. Agnolin[1,2,3], Ismar de Souza Carvalho[5], Makoto Manabe[6], Takanobu Tsujishi[6], Sebastián Rozadilla[1,2], Gabriel L. Lio[1] & Marcelo P. Isasi[1,2,3]

[1] Laboratorio de Anatomía Comparada y Evolución de los Vertebrados (LACEV) Museo Argentino de Ciencias Naturales "Bernardino Rivadavia" (MACN), Buenos Aires, Argentina; Av. Ángel Gallardo 470 (C1405DJR), Buenos Aires, Argentina.

[2] Consejo Nacional de Investigaciones Científicas y Técnicas (CONICET).

[3] Fundación de Historia Natural "Félix de Azara", Centro de Ciencias Naturales, Ambientales y Antropológicas, Universidad Maimónides, Hidalgo 775 (C1405BDB), Buenos Aires, Argentina.

[4] Museo Argentino de Ciencias Naturales "Bernardino Rivadavia" (MACN), Buenos Aires, Argentina; Av. Ángel Gallardo 470 (C1405DJR), Buenos Aires, Argentina.

[5] Universidade Federal do Rio de Janeiro, Av. Athos da Silveira Ramos, 274, Cidade Universitária, Ilha do Fundão, Rio de Janeiro RJ, 21941-916, Brazil; Centro de Geociências, Universidade de Coimbra, Rua Sílvio Lima, 3030-790 Coimbra, Portugal

[6] Department of Earth and Planetary Science, The University of Tokyo, 7-3-1 Hongo, Bunkyo-ku, Tokyo 113-0033, Japan.

*Corresponding author: cacopol@gmail.com (DP)

Brief title. New large peirosaurid from Patagonia

Abstract

The first crocodyliform specimen from the Maastrichtian Chorrillo Formation (Austral Basin, Patagonia) is here described. The discovery was made about 30 km to the SW of the town of El Calafate (Province of Santa Cruz, Argentina) and consists of a beautifully preserved and articulated skull and jaws, and part of the postcranial skeleton that was preserved encased in a large concretion. This new taxon belongs to the

notosuchian clade Peirosauridae, representing the latest and southernmost record for this group of crocodyliforms. The new taxon is recovered as closely related to other robust and broad-snouted peirosaurids that lived by the end of the Cretaceous Period, such as *Colhuehuapisuchus* from the Maastrichtian of Central Patagonia and *Miadanasuchus oblita* from the Maastrichtian of Madagascar. The completeness of the new specimen reveals, for the first time, the anatomy and body plan of a large and broad snouted peirosaurid. The new taxon bears large ziphodont teeth, a broad oreinirostral snout that is only slightly longer than 50% the skull length, and a deep adductor chamber in the temporal region and posterior mandibular ramus. CT scan study reveals a nasal cavity flanked by a large, but anteriorly restricted, antorbital sinus within the maxilla. The new crocodyliform completes the predatorial component of terrestrial ecosystems at high paleolatitudes by the end of the Cretaceous Period.

Key words: Crocodyliformes, Peirosauridae, Chorrillo Formation, Patagonia, Maastrichtian, Late Cretaceous

Introduction

The end of the Cretaceous Period is particularly well recorded in Patagonia [1, 2] In recent years a large collecting effort has resulted in a high number of new vertebrate records in different basins of Patagonia, including the discovery of new and diverse faunal associations in central [e.g. 3, 4] and southern [e.g., 5, 6, 7] Patagonia that complements previous knowledge from the Neuquén Basin in northern Patagonia [e.g.,

1, 8, 9, 10, 11]. Among the records from the Austral Basin, the most noteworthy are those from the Campanian Cerro Fortaleza [e.g. 6] and the Maastrichtian Chorrillo formations [e.g. 5, 12] in Argentina, and Cerro Dorotea Formation in southern Chile [e.g., 13, 7].

The Chorrillo Formation crops out in the SW corner of Santa Cruz Province, in Argentine Patagonia (Fig 1). These beds are particularly interesting as they have yielded a wide array of fossils from a Maastrichtian terrestrial ecosystem [5, 12], including pollen and spores [14], plant remains [15], freshwater and terrestrial invertebrates [16], fishes, frogs, and turtles. Dinosaurs recovered from these beds include the elasmarian *Isasicursor santacrucensis* [5, 17, 18], the large titanosaur *Nullotitan glaciaris* [5], the megaraptorid coelurosaur *Maip macrothorax* [18], the birds *Kookne yeutensis* and *Yatenavis iujensis* [5 19], as well as indeterminate parankylosaurs and hadrosaurids [17]. The vertebrate fossil assemblage also includes representatives of different mammalian clades, including monotremes, gondwanatherians, meridiolestidans, and therians [20, 21, 22].

Here, we expand the diversity of fossil vertebrates from the Chorrillo Formation with the description of the peirosaurid crocodyliform *Kostensuchus atrox* nov. gen. et sp., represented by an exquisitely preserved and articulated skeleton, lacking some of the limb bones and tail (Supporting Information S1: Fig S1–S2). This specimen is one of the best preserved and anatomically informative peirosaurid crocodyliform yet recorded, and the most complete representative of robust, broad snouted members of this clade. The new taxon was a large predator, approximately 3.5 meters long (estimation based on extrapolations with complete skeletons of *Caiman* and *Alligator*), second only to the megaraptorid theropod *Maip* (ca. 9 meters) [18] known from the Chorrillo Formation. *Kostensuchus* reveals the craniomandibular anatomy and body

plan of the large and robust peirosaurids that survived until the end Cretaceous in South America and Madagascar, and provides the southernmost record of this diverse crocodyliform clade. The record of *Kostensuchus* contributes to characterize the faunal association from the Maastrichtian of southern Patagonia (Austral Basin), and underscores the differences among coeval terrestrial ecosystems in central and northern Patagonia (Cañadón Asfalto and Neuquén basins).

Methods

Body mass was estimated based on dorsal cranial length (490 mm), following the approach of recent studies for notosuchian crocodyliforms [23] and the scaling equations based on extant crocodylians [24].

The affinities of *Kostensuchus* were tested using an expanded version taken from Fernández-Dumont et al. [25], which is in turn a modified version of datasets previously published [26, 27] in which we added the new taxon and increased the sample of peirosaurid crocodyliforms. The complete dataset includes 120 taxa scored across 444 characters and was analyzed using an equally weighted parsimony analysis in TNT v. 1.6 [28]. Tree searches consisted of using New Technology Searches until 100 hits to minimum length was achieved. A subsequent round of TBR branch swapping was applied on the most parsimonious trees obtained. Unstable taxa were detected using IterPCR [29, 30] and nodal support was evaluated using parsimony jackknife [31] considering the impact of unstable taxa [32].

All necessary permits were obtained for the described study, which complied with all relevant regulations.

Nomenclatural acts

The electronic edition of this article conforms to the requirements of the amended International Code of Zoological Nomenclature, and hence the new names contained herein are available under that Code from the electronic edition of this article. This published work and the nomenclatural acts it contains have been registered in ZooBank, the online registration system for the ICZN. The ZooBank LSIDs (Life Science Identifiers) can be resolved and the associated information viewed through any standard web browser by appending the LSID to the prefix ""http://zoobank.org/"".

The LSID for this publication is: XXXXXX

The electronic edition of this work was published in a journal with an ISSN, and has been archived and is available from the following digital repositories: PubMed Central, LOCKSS.

Results

Systematic Paleontology

Crocodyliformes Hay, 1930

Mesoeucrocodylia Whetstone and Whybrow, 1980

Peirosauria Leardi et al., 2024

Peirosauridae Gasparini, 1982

Kostensuchus atrox gen. et sp. nov.

Etymology. *Kosten*, refers to the Patagonian wind in Aonikenk language; and *suchus*, latinized from the Greek Souchos in references to the Egyptian crocodile-headed god (Sebek). The species epithet *atrox* means harsh in Greek.

Holotype. MPM-PV 23554 (Museo Provincial "Padre Molina", Río Gallegos, Santa Cruz, Argentina), articulated skull, jaws, cervical, dorsal and sacrals, rows of bony scutes along the vertebral sequence, cervical and dorsal ribs, scapular and pelvic girdles, complete right humerus and partial left humerus, and fragmentary remains of the hindlimbs.

Locality, horizon, and Age. Lower section of the Chorrillo Formation, at Estancia La Anita, approximately 30 km SW from El Calafate city (Fig 1). The specimen comes from a concretionary level exposed at the locality "Puma cave" (locality 4, 5), approximately 60 meters from the base of the Chorrillo Formation [12].

Diagnosis. *Kostensuchus* gen. nov. is among the largest known peirosaurids (dorsal skull length = 49 cm) diagnosed by the following combination of characters (autapomorphies marked by *): skull proportionally shorter, wider and higher than in other peirosaurids; rostrum comprising slightly over 50% the total skull length; sinusoidal alveolar margin of maxilla (shared with *Hamadasuchus*); completely ossified internarial bar; lacrimal lateral surface faintly ornamented as slightly depressed between antorbital fenestra and orbit*; jugal reaching the ventral end of a circular antorbital fenestra; subtrapezoidal external supratemporal fenestra, occupying close to 70% of skull roof width; absence of anterior floor of supratemporal fossa; flat dorsal surface of parietal in occipital view; distinct step on dorsal surface of the posterolateral process of squamosal; broad palatine anterior margin, forming a bread transversal suture with maxilla; paired parasagittal ridges on the ventral surface of the basisphenoid; convex

dorsal edge of surangular and craniomandibular articulation located at the level of dentary toothrow (shared with *Uberabasuchus*); ziphodont teeth; humerus with vertical orientation of insertion area of M. subscapularis above internal tuberosity; distal end of deltopectoral crest curves medially surpassing the lateromedial midpoint of humeral shaft; anterior surface of distal end of humerus separated from shaft by a distinct step forming a shelf.

Description and Comparisons.

Skull and jaws

In dorsal view the skull is trapezoidal-shaped due to the abrupt end of the rostrum (Fig 2). The supratemporal openings are visible only in dorsal view, and are located on the posterior half of the skull table. The lower jaw is robust and as deep as the skull at the level of the postorbital bar, indicating an extensive area for muscular attachment in the adductor chamber. The maximum length of skull is 49 cm taken from tip of the snout to the tip of mandibular retroarticular process (about 20% longer than in *Uberabasuchus* and *Hamadasuchus*). The maximum transverse width of the skull is at the level of the quadratojugals and the maximum height of articulated skull and jaws is located at the level of the anterior end of the postorbital and the anterior end of the external mandibular fenestra.

In lateral view the dorsal profile of the skull of *Kostensuchus* gen. nov. is almost straight (Fig 2) due to the strong dorsoventral height of the rostrum, as in *Gasparinisuchus* and *Hamadasuchus*, but differing from other peirosaurids (e.g., *Uberabasuchus*, *Montealtosuchus*) [33, 34] in which the snout dorsoventral height tapers rapidly towards the anterior end. Sutures among some cranial (e.g., frontal,

prefrontal, parietal) bones are difficult to discern due to the extensive pitted ornamentation that covers the external surface of the skull, a condition that is indicative of a fully grown ontogenetic stage (in agreement with the fused condition of the neural arches and centra of cervical vertebrae).

The external nasal apertures are elliptical and anterolaterally oriented, whereas the orbits face laterally. The antorbital fenestra is rounded and small, being approximately 25% the size of the orbits and is dorsoventrally aligned with the ventral margin of the orbit. The ventral margin of the snout is festooned, and teeth are restricted to the rostral half of the skull (ending anteriorly to the level of the orbit). The infratemporal fenestra is subtriangular, anteroposteriorly longer than dorsoventrally high, and about 66% the size of the obit. This fenestra faces laterodorsally, due to the lateral placement of the infratemporal bar relative to the upper temporal bar.

The rostrum is as dorsoventrally deep as transversely wide, conferring a general tubular aspect (Figs 2, 3). The lateral surface of the rostrum formed by the premaxilla and maxilla are subvertically oriented. This condition resembles that of *Hamadasuchus*, *Lomasuchus* and *Uberabasuchus*, but differs from *Montealtosuchus* and *Gasparinisuchus* in which the rostrum is dorsoventrally shallower relative to its width. The snout roughly represents slightly over 50% the length of the skull, so that the orbits are located just caudal to the mid-length of the skull. Other peirosaurids, including *Uberabasuchus* and *Montealtosuchus*, have a proportionately longer rostrum that occupies close to 60% of the total skull length. The surface of the snout is profusely ornamented, and only bears a tenuous longitudinal line that may correspond with the suture between right nasal and maxilla but the nasal-nasal suture cannot be traced.

The snout is transversely constricted at the level of the premaxilla-maxilla suture, forming a large and laterally open notch for the reception of the hypertrophied fourth dentary tooth. Anterior to this point the premaxilla has a large lateral bulge, caused by the alveolus of the hypertrophied fourth premaxillary tooth, and then narrows anteriorly to end in a pointed premaxillary process that meet the nasals forming a complete internarial bar. In dorsal view, the sharply pointed internarial bar contrasts with the transversely wide and curved arcade of the rostral margin of the paired premaxillae. The premaxillary component of the internarial bar is projected anterolaterally forming an angle of approximately 60 degrees with the alveolar margin and surpasses anteriorly the level of the first premaxillary tooth. A large and complete internarial bar is present in *Uberabasuchus* but their presence in other peirosaurids cannot be confirmed due to incompleteness of this delicate part of the skull (e.g., *Montealtosuchus*, *Peirosaurus, Lomasuchus*). This septum, however, is certainly absent in most other notosuchian clades, including baurusuchids and sphagesaurians. *Kostensuchus* gen. nov. and *Uberabasuchus* share a similarly rostrally arched internarial bar, although in *Uberabasuchus* the snout is more anteriorly projected than in *Kostensuchus* gen nov.

The posterior and ventral margins of the narial openings are surrounded by a broad and smooth perinarial fossa that extends ventrally reaching the alveolar margin along the level of the first two premaxillary teeth. The presence of a perinarial fossa is a widespread feature among notosuchians, but the large fossa reaching the alveolar margin of *Kostensuchus* gen. nov. resemble in size and extension only that of baurusuchids and peirosaurids, such as the one present in *Uberabasuchus*, *Hamadasuchus*, and *Peirosaurus*. At the posterodorsal corner of the narial opening the premaxilla bears a notch and and a small shelf that overhangs the posterodorsal region

of the perinarial fossa. The right perinarial fossa bears a single and large neurovascular foramen, present also in *Uberabasuchus* and *Hamadasuchus*. The presence of this foramen cannot be determined with certainty on the left side due to partial damage.

The maxilla of *Kostensuchus* gen. nov. has a festooned alveolar margin, ventrally extended at the level of the third maxillary tooth, as in other peirosaurids, and a smaller posterior ventral outgrowth at the level of the antorbital fenestra. The latter is also present in *Hamadasuchus* but absent or much smaller in other peirosaurids (e.g., *Uberabasuchus*, *Lomasuchus*, *Montealtosuchus*). In *Kostensuchus* gen. nov. the lateral surface of maxilla exhibits (on both sides) a longitudinal and sigmoid groove, extending backwards from the posterior margin of the nasal aperture. It runs approximately parallel to the alveolar margin of maxilla, describing a ventral curvature behind the notch for the mandibular caniniform. This groove separates two different decoration patterns on the lateral surface of the snout: above the groove predominate large pits closely positioned to each other, and below the groove the pits are much smaller. *Uberabasuchus* and *Montealtosuchus* also exhibit such a longitudinal and sigmoid groove, although it seems to be located more ventrally than in *Kostensuchus* and does not seem to limit different types of surface ornamentation. *Lomasuchus* clearly have two different types of ornamentation on the maxillary surface, but the groove seems to be much more subtle than in *Kostensuchus* gen. nov. and *Uberabasuchus*. It is not clear the anatomical significance of such a groove, but it approximately follows the course dorsal end of the alveoli and the course of the paired maxillary artery and vein that runs within the maxilla [35], whereas the above-mentioned groove runs on the external surface of the maxilla. Although with some variation, this feature seems to be so far exclusive of South American peirosaurids as it is absent in *Hamadasuchus* and mahajangasuchids. CT scan of the skull of *Kostensuchus* gen. nov. revealed that the maxilla lacks recesses

within the palatal branch of the maxilla and the postvestibular sinus [36] but it has a large internal antorbital sinus that extends anteroventrally from the antorbital fenestra. The maxilla forms the entire anterior margin of the antorbital fenestra and lacks a well-developed antorbital fossa, although a small surface at the anteroventral corner of the antorbital fenestra is smooth. The nasals of *Kostensuchus* gen. nov. are heavily ornamented and their sutural contacts cannot be clearly determined except for their suture with the premaxilla and maxilla at the anterior region of the rostrum.

The jugal external surface is flat, dorsoventrally high below to orbit, and heavily ornamented as in other peirosaurids. The jugal extends anteriorly to the orbit and almost reaches the posteroventral corner of the antorbital fenestra, resembling the condition of other peirosaurids (*Uberabasuchus*, *Montealtosuchus*, *Hamadasuchus*). The jugal dorsoventral depth decreases gradually posterior to the orbit and has a long overlapping contact with the quadratojugal along which it tapers to a pointed end located close to the craniomandibular articulation. The ascending process of the jugal is superficial at its anterior end, rather than inset relative to the external jugal surface as in neosuchians. The posterior margin of the ascending process, however, in inset relative to the lateral surface of the jugal, a condition also present in other peirosaurids (*Uberabasuchus*, *Montealtosuchus*, *Hamadasuchus*), *Kaprosuchus*, and some uruguaysuchids (*A. patagonicus*, *A. wegeneri*). This process is robust and cylindrical and laterally overlaps the descending process of the postorbital. The lacrimal external surface is laminar and the central part of its lateral surface bears a fainter ornamentation than the rest of the rostrum between the orbit and the antorbital fenestra . This faintly ornamented surface is slightly depressed relative to the dorsal and ventral ends of the lacrimal. *Hamadasuchus*. This morphology differs from that of other South American peirosaurids (e.g., *Uberabasuchus*, *Montealtosuchus*) and uruguaysuchids, which have a

well-developed vertical ridge on the lacrimal that separates a smooth and deeply depressed antorbital fossa from the posteriorly located ornamented surface of the lacrimal. It also differs from the condition of *Hamadasuchus* that lacks a lacrimal antorbital fossa and has its entire lateral surface as ornamented as the rest of the rostrum. The dorsal end of the lacrimal bears an ornamented bulge located close to the orbital margin, at its contact with the prefrontal and the anterior palpebral. A similar but less developed bulge is present in *Montealtosuchus* and *Uberabasuchus* but not in *Hamadasuchus*.

The sutural contacts of the prefrontal with other elements of the skull are difficult to discern but its posterior contact with the frontal seems to be located at the anteroposterior midpoint of the orbit. From this point forward the prefrontal constraints the width of the frontal and reaches the posterior end of the nasal. The lateral margin of the prefrontal is completely bounded by a large and triangular shaped anterior palpebral that overhangs the orbital opening, covering its anterodorsal region. The anterior palpebral is broadly sutured to the frontal and to the posterior palpebral. The posterior palpebral is shorter than the anterior palpebral and is also triangular shaped but with its base facing anteriorly and its pointed apex directed posteriorly. Its anterior flat margin is strongly sutured to the anterior palpebral and its medial margin is sutured to the postorbital. The posterolateral end of the posterior palpebral overhangs the orbit at the level of the descending process of the postorbital. This configuration of the palpebrals resembles that of other peirosaurids (*Montealtosuchus, Lomasuchus, Uberabasuchus*).

The frontal does not participate from the external margin of the orbit due to the presence of the large palpebral[illegible]he dorsal surface of the frontal is triangular shaped, flat, and heavily ornamented and lacks any signs of a sagittal crest or elevated orbital rims. The interorbital region of the frontal is broad relative to its posterior end, which

forms part of the anteromedial margin of the external supratemporal fenestra, as in other peirosaurids (*Lomasuchus*, *Uberabasuchus*, *Montealtosuchus*, *Rukwasuchus*) but not in *Hamadasuchus*.

In *Kostensuchus* the temporal region is anteroposteriorly longer relative to the skull length than in other peirosauruids, such as *Uberabasuchus* or *Lomasuchus* that have a larger rostrum and a posteriorly displaced temporal region. The external supratemporal fenestra of *Kostensuchus* gen. nov. are broader posteriorly than anteriorly as in other peirosaurids. The supratemporal fossa bears a large posterior floor formed by the parietal and squamosal but lacks a development of a frontal-postorbital floor at the anterior end, differing from the anterior floor of the supratemporal fossa in other peirosaurids (*Montealtosuchus*, *Uberabasuchus*, *Lomasuchus*, *Hamadasuchus*). with an extensive posterolateral projection that creates a relatively narrow upper temporal bar (formed by the squamosal and postorbital). The width of this surface is approximately 30% the lateromedial width of the external supratemporal fenestra, as in *Uberabasuchus* and *Hamadasuchus*, differing from the much broader condition of the postorbital-squamosal bar in *Montealtosuchus* and *Lomasuchus*. The parietal is a single element, as in other crocodylomorphs [37], and its dorsal surface is flat and deeply ornamented. The parietal width between the supratemporal fenestra is approximately 30% the width of each of these openings, resembling the condition of *Lomasuchus* but differing from both the proportionately wider parietal bar of *Uberabasuchus* and *Montealtosuchus* and the much narrower parietal bar of baurusuchids. The parietal dorsal surface is flat in occipital view as in *Gasparinisuchus* and *Hamadasuchus*, lacking the distinct central concavity present in other peirosaurids (*Montealtosuchus*, *Uberabasuchus*, *Lomasuchus*, *Rukwasuchus*).

The postorbital has a narrow exposure on the skull roof and has an oblique anterolateral margin, as in all notosuchians. This margin is sutured to the posterior palpebral and the postorbital-squamosal contact is an oblique suture located at the anteroposterior midpoint of the supratemporal fenestra. The descending process of the postorbital is inset relative to the skull rook, as in all mesoeucrocodylians. The lateral surface of the descending process is deeply concave because the otic recess extends up to the anterior end of the postorbital, as in other notosuchians. This concave smooth surface faces posterolaterally and contacts the quadrate and quadratojugal along its posterior margin, and the ascending process of the jugal at its anteroventral end. The squamosal forms the posterolateral corner of the skull roof and has a long posterolateral process that reaches the anteroposterior level of the craniomandibular articulation. The length of the posterolateral process resembles the condition of some peirosaurids (*Lomasuchus, Uberabasuchus, Montealtosuchus*) but is longer than in *Hamadasuchus* and *Rukwasuchus*. The dorsal surface of the posterolateral process is ornamented and bears a subtle step located at its midpoint, so that the posterior region of the posterolateral process is ventrally recessed relative to the dorsal surface of the skull roof, differing from other peirosaurids that lack this step. The posterolateral process of the squamosal is straight and slightly ventrally deflected (as in *Hamadasuchus*), but not as much as in *Lomasuchus*, *Uberabasuchus*, and *Montealtosuchus*. The condition of all peirosaurids however differs from the condition of baurusuchids (e.g., *Baurusuchus*, *Pissarrachampsa*) in which this process is strongly arched and ventrally projected, almost reaching the same level as the quadrate condyles. Within the otic recess the squamosal forms the dorsal half of the otic aperture and has an extensive sutural contact with the quadrate posterior to the otic aperture. The otic aperture is subrectangular with an angled corner at its anterior margin, as in other peirosaurids. The quadrate forms the

ventral half of the otic aperture and bears a small foramen just anterior to the otic aperture, referred as the subtympanic foramen [*sensu* 38]. This is the only accessory foramen of the otic recess, resembling the condition of other peirosaurids, sebecids, and uruguaysuchids, but differing from the elliptical openings present in some notosuchians (e.g., *Notosuchus*), baurusuchids, and more basal crocodyliforms (e.g., *Protosuchus*, *Burkesuchus*). The articular region of the quadrate is relatively short and broad and forms together with the quadratojugal a robust craniomandibular articulation. A small siphoneal foramen is present on the posterior surface of the quadrate, just above the medial articular condyle. The quadratojugal has a large ornamented posteroventral region and a narrower and smooth ascending process that forms the posterior margin of the infratemporal fenestra. The anterior process of the quadratojugal is absent so that this bone fails to form the ventral margin of the infratemporal fenestra, as in other peirosaurids.

The parietals and squamosals form a continuous occipital ridge that projects posteriorly as a slightly developed nuchal crest. This crest is slightly dorsally convex in occipital view as the lateral region of the squamosal is slightly deflected ventrally. The occipital surface formed by the squamosal and otoccipitals is subrectangular in shape and almost as deep as wide.

The lower jaw of *Kostensuchus* is dorsoventrally deep, especially at its posterior end which is slightly deeper than the posterior half of the skull (Fig 2). The jaw is stouter than in other peirosaurids. The symphyseal region is transversely broad (Fig 4), being its width 65% than its length (including the splenial participation in the symphysis). This represents the broadest mandibular symphysis among all peirosaurids in which broad snouted forms have width/length ratios close to 60% (e.g., *Colhuehuapisuchus*, *Gasparinisuchus*, *Barrosasuchus*, *Miadanasuchus*) and narrow

snouted peirosaurids have ratios ranging between 40% and 50% (*Montealtosuchus, Uberabasuchus, Patagosuchus, Bayomesasuchus*). An event narrower symphysis is present in *Hamadasuchus* (ranging 30%) and certainly in the long snouted itasuchids (*Itasuchus, Pepesuchus*). As in other broad snouted peirosaurids, the rostral margin of the dentaries anterior the caniniform meet at a broad angle (approximately 140 degrees). The dentary is bulged at the level of the caniniform and constricted posteriorly to it, forming a lateral concavity where the first enlarged maxillary caniniform occludes (Fig 4). This constriction is present in most peirosaurids, except for *Colhuehuapisuchus* in which the dentaries are almost straight posterior to the lower caniniform (which in turn would indicate a less developed maxillary caniniform than in *Kostensuchus* gen. nov.).

The dentary is the longest bone of the lower jaw, representing twice the length of the angular plus retroarticular. In lateral view the dentary tapers gradually towards its anterior end as in other peirosaurids, uruguaysuchids, and sphagesaurians, but differs from the strongly angled profile of baurusuchids and some sebecoids (e.g., *Iberosuchus, Bretesuchus*). The lateral surface shows a change in ornamentation pattern: along the anterior half, the surface is ornamented with elliptical pits of small size whereas the posterior half is gradually shifts to an ornamentation composed by a complex pattern of oblique ridges and grooves (close to the mandibular fenestra). The posterodorsal region of the dentary bears a broad longitudinal sulcus below the buccal margin, a feature shared with *Uberabasuchus* and *Montealtosuchus*. The splenial forms part of the mandibular symphysis, but this contribution in ventral view is less than 20% of the symphyseal length. In both ventral and dorsal views, the splenial-dentary suture is transversely oriented, as in the broad snouted *Colhuehuapisuchus* and *Miadanasuchus*, rather than V-shaped as in other peirosaurids. The posterior end of the splenial-splenial suture bears a posterior peg, a common feature with several peirosaurids and other

notosuchians. The splenial bears a notably large slot-like foramen intermandibularis oralis posterior to the symphyseal region, a feature that is also present in the broad snouted *Colhuehuapisuchus* and *Miadanasuchus.* The splenial is broadly exposed on the ventral surface of the mandibular ramus along its contact with the dentary and angular. The medial surface of the splenial is only exposed along its posterior region, which is flat, smooth, and completely sutured with the angular, denoting the absence of a foramen intermandibularis caudalis as in all notosuchians.

The angular and surangular are also ornamented along their lateral surface, with pits being more frequent at its posterior end and ridges more frequent anteriorly (especially in the surangular). The angular is sutured to the dentary by an oblique suture that runs posteroventrally from the acute anterior end of the external mandibular fenestra. The dorsal margin of the angular forms the complete ventral margin of the external mandibular fenestra. The angular-surangular suture runs posteriorly and slightly ventrally from the posterior end of the mandibular fenestra, so that most of the lateral surface of the mandibular ramus is formed by the surangular at the level of the craniomandibular articulation. The ventral margin of the angular is slightly curved dorsally towards its posterior end and bears a very low and broad angular ridge at the level of the external mandibular fenestra. This contrasts with the sharper and more prominent angular ridge of *Uberabasuchu*s and *Montealtosuchus* that extends posteriorly surpassing the level of the craniomandibular articulation.

The anterior end of the surangular is forked at its anterior contact with the dentary. The ventral margin of the surangular has a lineal overlapping suture with the dentary and this contact reaches the posterodorsal corner of the external mandibular fenestra. The surangular forms the rounded posterior edge of this opening and is sutured to the angular at the posteroventral corner of this fenestra. The posterior region of the

surangular bears a broad longitudinal ridge that extends posteriorly to the level of the glenoid facet and borders the anterior region of the retroarticular process (Fig 2), as in other peirosaurids (e.g., *Uberabasuchus*, *Montealtosuchus*). The surangular forms most of the lateral flange of the retroarticular process. This surface is triangular, dorsally concave, and is separated from the surangular contribution to the glenoid facet by an elevated transversal ridge. The articular forms part of the lateral flange of the retroarticular process and bears a broad and low longitudinal ridge that separates the medial from the lateral flange. This ridge ends posteriorly in a rounded bulge that is directed posterodorsally (as in *Montealtosuchus* but not as dorsally recurved as in *Uberabasuchus*). The medial flange of the retroarticular process extends ventromedially and its slightly concave surface faces posteromedially. The ventral margin of this flange is convex as other peirosaurids and uruguaysuchids, but not as extended and paddle shaped as in sphagesaurians and baurusuchids. The medial flange bears the foramen aerum and a small bulge located close to it (as in uruguaysuchids and neosuchians). The articular forms the medial half of the glenoid facet for the quadrate condyles, which faces dorsally and is separated from the retroarticular process by an elevated transversal buttress.

Dentition

Kostensuchus gen. nov. exhibits a morphological pattern of the tooth row that resembles that of other peirosaurids, consisting in conical teeth anterior to the large mandibular caniniform, and post-caniniforms that are leaf-shaped and smaller in size (Fig 2). The upper toothrow has enlarged caniniforms in the premaxilla and the maxillary and bears a tooth count of 15 elements, which is smaller than that of other peirosaurids (e.g.,

Hamadasuchus, Lomasuchus, Montealtosuchus). All preserved teeth bear small symmetrical denticles along the entire mesial and distal margins, characteristic of a fully ziphodont dentition.

The premaxilla bears five teeth as in most peirosaurids , the two anteriormost of which are small and closely positioned to each other. The fourth tooth is the largest, followed by the third that is 70% and the fifth one is approximately 50% the size of the largest element (considering the basal mesiodistal width of the crown). The upper toothrow has a long gap between the premaxillary and maxillary dentition that accommodates a large dentary caniniform. The right maxilla has ten teeth exposed on the lateral surface. The first three increase progressively their size up to the third maxillary tooth, which is the largest tooth of the upper row and similar in size to the lower caniniform. The following five maxillary teeth are notably smaller. The posteriormost teeth are short-crowned, lanceolate, and much more buccolingually compressed than the preceding conical-shaped teeth. The change in shape and relative sizes of the teeth in *Kostensuchus* gen. nov. closely resemble that of *Uberabasuchus and Montealtosuchus,* but differs from *Hamadasuchus* in the absence of a second enlarged maxillary in its posterior region. All peirosaurids nonetheless fall within the generalized condition of bearing a large tooth count, compared with baurusuchids or sphagesaurids that have a maxillary tooth count limited to five or six teeth.

The dentary teeth are less well exposed in *Kostensuchus* gen. nov. but CT data, as well as the natural fracture present along the symphyseal region allows determining the procumbent nature of the two anteriormost dentary teeth (Fig 3), as in *Colhuehuapisuchus* but differing from other peirosaurids (e.g., *Montealtosuchus, Uberabasuchus*) in which these teeth are not directed as anteriorly as in these two Patagonian taxa. The fourth dentary tooth is more than twice the length and height of

other lower teeth and strongly protrudes dorsally surpassing the dorsoventral midpoint of the snout. The post-caniniform mandibular teeth are not exposed but CT data shows they have a posterior wave of minor size variation.

Postcranial skeleton

The postcranial skeleton of *Kostensuchus* gen. nov. is represented by the presacral and sacral regions of the vertebral column as well as a large part of the dorsal shield of osteoderms, scapular girdle, left humerus and partial remains of the right humerus, ilia, and ischia (Fig 5; Supporting Information S1: Fig S1–S2).

The cervical series has relatively low anteriormost cervicals that progressively become anteroposteriorly short and dorsoventrally high (Fig 6). The posterior cervicals have high rod-like neural spines, which are as high as the rest of the vertebrae. This feature is also present in *Uberabasuchus*, *Montealtosuchus*, and is also present in some sphagesaurians (e.g., *Notosuchus*) and baurusuchids. The prezygapophyses are directed anterodorsally and bear a straight anterior margin but the postzygapophyses are occluded by sediment or the overlying osteoderms. The cervical centra are poorly constricted and bear a knob like hypapophysis. The cervical ribs are anteroposteriorly elongated as in all crocodyliforms and bear a distinct knob at the base of the posterior process, which is much more reduced than the well-developed process described for *A. tsangatsangana* and *Mahajangasuchus* [39].

The dorsals have a much lower neural spine and a proportionately longer and lower centrum and neural arch (Fig 7). The parapophysis migrates gradually along the first five dorsal elements from the dorsal end of the centrum to the same level of the

diapohysis. The dorsal neural spines are low and have a convex anterior and concave posterior margin.

The sacrum is composed of two vertebrae, although the absence of a caudal series precludes determining the presence of a caudosacral element, as in *A. gomesii*. The sacrum was preserved in articulation with the last dorsal vertebrae and with the left ilium. The first sacral rib attaches to the ilium at the level of the preacetabular process and is much shorter anteroposteriorly than the second sacral rib. The second sacral rib is wing=-shaped and extends posterolaterally exceeding the posterior end of the centrum and neural arch (including the postzygapophyses) of the second sacral vertebra.

The scapulae of *Kostensuchus* gen. nov. are preserved on both sides of the body, close to their original anatomical position (Fig 8). It is dorsoventrally short and anteroposteriorly broad in comparison with other crocodyliforms. In general terms both peirosaurids and uruguaysuchids have a relatively short and broad scapula, but in the case of *Kostensuchus* gen. nov. this feature is even more notable. The dorsal blade of the scapula is approximately 90% of the maximum dorsoventral height of the scapula. The constriction between the ventral and dorsal expansions is limited, being approximately 75% the anteroposterior width of the ventral end of the scapula. The glenoid articular facet of the scapula faces ventrally, rather than posteroventrally as in *Mahajangasuchus*. This region is lateromedially thick, whereas dorsal to the central constriction the scapula becomes laminar (throughout the scapular blade). The coracoid is approximately as long as the scapula and lacks signs of a torsion along its shaft. The coracoid foramen is dorsoventrally elongated and located centrally on the proximal expansion. The glenoid facet faces posterodorsally and its ventral end bears a well-developed lip so that the region just ventral to the glenoid is markedly concave, as occurs in baurusuchids [27].

The humerus (Fig 9) is remarkably robust and distally expanded in comparison with that of other notosuchians and other crocodyliforms. The width of the proximal end is approximately 30% of the humeral length, which does not differ markedly from the range of other taxa. The width of the distal end, however, is 30% wider than the proximal end and 40% of the total humeral length. The proximal end of the deltopectoral crest is damaged but it can be determined that the crest is triangular shaped in lateral view and bears a rounded tubercle at its tip. Distal to the tubercle the crest extends distomedially onto the shaft and surpasses the lateromedial midpoint of the shaft, as in baurusuchids and sebecids [40]. The expanded distal end bears two prominent condyles and flat lateral and medial surfaces bounded by sharp supracondylar crests. The posterior surface of the humerus bears and shallow fossa between the posterior supracondylar crests. However, the anterior surface of the distal humerus bears a deep rounded fossa between the supracondylar crests (presenting a morphology not recorded in other crocodyliforms but that may characterize at least some peirosaurids). Distal to this fossa, the anterior surface of the humerus bears a distinct step and a proximally facing shelf that extends lateromedially along the entire width of the humerus. This feature has only been noted in *Sebecus icaeorhinus* and *Iberosuchus* but is absent in baurusuchids and in *Mahajangasuchus* [40].

Both ilia of *Kostensuchus* gen. nov. are available: the left one preserved in articulation with the sacrum. The ilium has a well-developed preacetabular process (Fig 10) in comparison with other notosuchians, nonetheless, this process is not as long as in more basal crocodyliforms (e.g., *Protosuchus*). The acetabulum resembles the condition of uruguaysuchids (e.g., *A. gomesii*) in that it is deeper than in neosuchians but lacks the robust and horizontally directed supraacetabular crest that forms a thick acetabular roof in sphagesaurians (e.g., *Notosuchus*), baurusuchids (e.g., *Baurusuchus albertoi*), and to

a lesser degree sebecids [40]. The insertion area of the *Mm. iliotibialis* forms a rugose area along the supraacetabular crest at the level of the posterior half of the acetabulum. This scar does not extend anteriorly towards the preacetabular process as in *Sebecus icaeorhinus*. The pubic peduncle is notched, forming deep and narrow incisure with an angle of approximately 30 degrees that resembles that of *Mahajangasuchus* but differs from that of neosuchians or other notosuchians (e.g., *Sebecus*, *Notosuchus*) that have a much broader angle and shallower incisure. The ischiadic peduncle is broader than the pubic peduncle and bears a flat antitrochanter on its acetabular surface. The postacetabular process is longer than the acetabulum and is dorsoventrally high, being as deep as the acetabulum. The posterior end of the postacetabular process is dorsoventrally deep, with a horizontal ventral margin, and a subrectangular posterior end (as in most notosuchians, except for *Sebecus icaeorhinus*). The ischium is more expanded at its ventral end than at its dorsal (acetabular) region, as in *Mahajangasuchus* but differing from much less expanded ventral end of baurusuchids (*Stratiotosuchus*) and neosuchians. The proximal end contacts the pubic and ischiadic peduncle of the ilium and forms the ventral end of the acetabulum, excluding the pubis from it, as in all mesoeucrocodylians.

Phylogenetic affinities

Peirosaurids are recorded in South America, Africa, and Madagascar and have been recognized as important components of Cretaceous faunas of Gondwana for the last 40 years [41, 42]. Currently, there is ample consensus in their monophyly [43] and most phylogenies depict the African *Hamadasuchus* as sister group of South American peirosaurids [44; 45; 25; but see 46 for an alternative arrangement]. However, at the

moment there is a lack of a stable resolution in the internal relationships of Peirosauridae. Several authors have noted the presence of peirosaurids with broad jaws [47, 48, 49, 50] but most of them are known from fragmentary mandibular remains. The only exception is *Gasparinisuchus* [47] that is known from a specimen with partially preserved craniomandibular remains (previously referred to *Peirosaurus*) [51]. Although broad-snouted peirosaurids are potentially closely related to each other, so far, these taxa have been rarely included in phylogenetic analyses, likely due to the incompleteness of their remains. The discovery of *Kostensusuchus* with an exquisitely preserved skull allows understanding for the first time this morphological type as well as testing the relationships of broad snouted peirosaurids within the context of other members of this clade.

Our phylogenetic results (Fig 11; see Supporting Information S1) support a monophyletic Peirosauridae as in previous analyses [26, 25, 45] but also retrieves a clade of broad snouted peirosaurids, composed by the Late Cretaceous taxa *Kostensuchus* gen. nov., *Colhuehuapisuchus*, *Miadanasuchus*, and *Gasparinisuchus*. Within this group, *Gasparinisuchus* and *Colhuehuapisuchus* are depicted as sister taxa due to the presence of a straight alveolar margin of the dentary posterior to the lower caniniform (in dorsal view) rather than the markedly concave margin of other peirosaurids (including *Kostensuchus* gen. nov.). *Kostensuchus* gen. nov. is depicted as the sister taxon of this clade, forming a group that is diagnosed by the presence of procumbent anterior mandibular alveoli (not preserved in *Gasparinisuchus*). The clade of broad snouted peirosaurids is formed with the addition of *Miadanasuchus oblita* [50, 52] from the Maastrichtian of Madagascar. This group is diagnosed by the presence of broad U-shaped symphyseal region of the dentaries (in ventral view), the presence of a large slot-like foramen intramandibularis oralis facing medially on the splenial, and a

transversely oriented splenial-dentary suture at the mandibular symphysis (on both the ventral and dorsal surface).

The clade of South American peirosaurids is retrieved as monophyletic, leaving the African *Hamadasuchus* as the earliest diverging lineage of Peirosauridae. The South American clade is diagnosed by the presence of the frontal participation on the internal supratemporal fenestra preventing the parietal-postorbital contact, a wedge-like process of the maxilla on the lateral surface of the premaxilla-maxilla suture, and the palpebrals extensively sutured to each other and to the lateral surface of the frontal. Peirosauridae is diagnosed by the presence of true ziphodont teeth (rather than pseudoziphodont as in mahajangasuchids), nasal-maxilla sutures nearly parallel to each other, basisphenoid with long dorsolateral extension exposed on the lateral surface of the braincase, large perinarial fossa facing anteriorly toward the alveolar margin, foramen in perinarial depression, palatine suture with maxilla transversal and slight invaginated, and prominent depression on palate near alveolar margin at the level of the sixth or seventh alveolus. Further phylogenetic data can be found in the Supporting Information S1.

Support values for most nodes are low due to the presence of fragmentary taxa and most high-level nodes within Mesoeucrocodylia are collapsed (see Supporting Information S1: Fig. S5), but Peirosauridae are among the lower-level clades retrieved with jackknife frequencies higher than 50%, along with Sphagesauria, Mahajangasuchidae, Atoposauridae, Thalattosuchia, Tethysuchia, Goniopholidae, Eusuchia. The impact of fragmentary taxa that became unstable in parsimony jackknife support analysis is evident when the *pcrjak* method [32] is used, which ignores the impact of these wildcards on support values. The *pcrjak* tree retrieves most major nodes within Crocodyliformes with high support (see Supporting Information S1: Fig. S6). In this tree the support is high also for Peirosauridae (80%) and for the South American

clade of peirosaurids (71%). Furthermore, the close affinity of the broad snouted *Kostensuchus* gen. nov. and *Colhuehuapisuchus* is supported with 79%, given that both *Miadanasuchus* and *Gasparinisichus* are detected as unstable during the jackknife replicates.

Discussion

Broad-snouted peirosaurids as apex predators. The discovery of *Kostensuchus* gen. nov. provides the first comprehensive insight into the anatomy of large-bodied, broad-snouted peirosaurids that thrived at the end of the Cretaceous. Represented by a nearly complete skeleton (Fig 5), *Kostensuchus* gen. nov. contrasts sharply with the fragmentary remains of other Maastrichtian broad-snouted peirosaurids, such as *Colhuehuapisuchus* and *Miadanasuchus*, which are known only from partial mandibles. *Kostensuchus atrox* gen. et sp. nov. is the largest peirosaurid for which a reliable size estimate can be calculated, with an estimated body length of 3.5 meters and a body mass of 250 kg (see Materials and Methods). This body mass is significantly greater than those of earlier narrow-snouted peirosaurids, whose body mass estimates range between 12 and 63 kg (e.g., *Montealtosuchus*, *Uberabasuchus*, *Lomasuchus*, *Hamadasuchus*) [23]. Thus, peirosaurids appear to align with a broader trend observed in some notosuchian clades, which evolved into larger body sizes over the course of the Cretaceous [53]. This shift has been linked to a transition from omnivorous, medium-sized forms to larger, hypercarnivorous apex predators [54, 55].

Broad-snouted peirosaurids were likely top predators, as evidenced by several adaptations indicative of a predatory lifestyle and its large body size. For instance, *Kostensuchus* gen. nov. had a broad, robust snout that comprised slightly over 50% of

its total skull length, a large adductor chamber in the skull, and a deep mandibular adductor fossa, suggesting the presence of powerful temporal muscles. This morphology, combined with its exceptionally large conical teeth along the anterior and mid regions of the toothrow, with labiolingually broad teeth with ziphodont margins, results in a lengthening of the shearing edges whose primary functions are puncturing and slicing through the flesh of sizable prey. These features have been interpreted as adaptations towards hypercarnivory in baurusuchid crocodyliforms and other large predators capable of subduing large struggling prey [54, 55, 56, 57]. This morphology, combined with its exceptionally large, labiolingually broad conical teeth with ziphodont margins, indicates a capacity for generating strong bite forces capable of subduing sizable prey. Notably, the teeth of *Kostensuchus* gen. nov. are approximately twice the size of equivalent teeth in narrow-snouted peirosaurids, such as *Montealtosuchus* or *Hamadasuchus*, in both apicobasal height and mesiodistal length [see 58]. This anatomical evidence supports the interpretation that broad-snouted peirosaurids evolved into hypercarnivores by the end of the Cretaceous, increasing in body size and developing adaptations for hunting larger prey, likely medium-sized tetrapods such as ornithischian dinosaurs (based on the faunal assemblage of the Chorrillo Formation). *Kostensuchus* gen. nov. emerges as a large predator among its kin and within the Maastrichtian Chorrillo Formation, surpassed in size only by the megaraptorid theropod *Maip*. The discovery of *Kostensuchus* significantly enriches our understanding of the terrestrial ecosystems that developed along the floodplains of a freshwater setting under a temperate to warm, seasonally humid climate at high latitudes in Patagonia [12, 14, 15, 59].

In the context of South America, broad-snouted peirosaurids are currently known from the latest Cretaceous at high-latitude regions, including the newly

described *Kostensuchus* gen. nov. from the Chorrillo Formation and *Colhuehuapisuchus* from the Colhue Huapi Formation [49]. These formations have similarities in their predator guild composition, with broad-snouted peirosaurids occupying a role alongside megaraptoran theropods as apex predators [18, 60]. The growing understanding of vertebrate assemblages in these southern Patagonian ecosystems from the end of the Cretaceous highlights increasing differences compared to the better known faunas from other regions of South America, such as northern Patagonia and Brazil. In these other regions, predator guilds seem to have been dominated by abelisaurids, with no evidence of megaraptorans [5, 18]. Additionally, large-bodied broad-snouted peirosaurids, such as *Kostensuchus*, have not been identified in these areas despite decades of intensive sampling in northern Patagonia and southern Brazil. The absence of megaraptorans and broad-snouted peirosaurids in these regions at the end of the Cretaceous might be indicative of distinct ecological settings compared to those in southern Patagonia. The causes behind these potential regional differences remain poorly understood. Future research may shed light on whether these differences were driven by environmental factors, competitive interactions, or a combination of both.

Broad-snouted peirosaurids and baurusuchids. The discovery of *Kostensuchus* and the completeness of its remains allow us to compare its anatomy with that of the other group of large predatory crocodyliforms from the Cretaceous of South America: Baurusuchidae [54, 61, 62]. These two clades of hypercarnivorous crocodyliforms share certain cranial and dental features related to their feeding habits, yet they also have structural differences in these anatomical regions. The dentition of *Kostensuchus* and baurusuchids share the presence of notably large, conical teeth that are almost as broad buccolingually as long mesiodistally, with small serrations along their entire mesial and distal margins. Both groups have a similar pattern of size variation in their dentition,

including an enlarged premaxillary tooth, a hypertrophied maxillary tooth, and a similarly large fourth dentary tooth. While this pattern is not unique to these groups, the relative size of their large teeth (both in mesiodistal length and apicobasal height compared to skull size) is greater than in other notosuchians, with the exception of *Kaprosuchus* [illegible]].

Kostensuchus gen. nov. and baurusuchids differ in several cranial and postcranial features. In the dentition, two notable differences are the presence of procumbent anterior dentary teeth in *Kostensuchus* gen. nov. (a trait not observed in baurusuchids or other peirosaurids) and a higher number of maxillary teeth, with *Kostensuchus* gen. nov. having over ten compared to the reduced number of five maxillary teeth in baurusuchids. In terms of skull proportions, baurusuchids (as well as many narrow-snouted peirosaurids) have a rostrum that is longer than the rest of the skull whereas *Kostensuchus* gen. nov. has a broad rostrum that is proportionately shorter. Additional differences in rostral morphology include the deeper and narrower proportions of the baurusuchid snout. This is especially noticeable in dorsal view, where the profile of the skull gradually tapers anteriorly in peirosaurids (including *Kostensuchus* gen. nov.), whereas in baurusuchids the snout lateral margins are parallel to each other and the skull broadens abruptly near the orbits. The anterior (symphyseal) region of the lower jaws in *Kostensuchus* gen. nov. is lateromedially broader and dorsoventrally lower compared to baurusuchids. Differences are also present in the posterior region of the mandibular ramus, where the adductor muscle attachments are located. In *Kostensuchus* gen. nov. and other peirosaurids, the external mandibular fenestra is small and low, and the angular has a low crest along its ventral margin. In baurusuchids, the external mandibular fenestra is larger, and the insertion of the *M. pterygoideus anterior* extends onto the lateral surface of the angular.

The well-preserved humerus of *Kostensuchus* gen. nov. allows for a detailed comparison with baurusuchids, revealing both similarities and differences in their forelimb anatomy. A notable similarity is the presence of a distomedially extended deltopectoral crest on the humerus of both *Kostensuchus* gen. nov. and baurusuchids. This shared feature suggests that both groups had powerful forelimbs, likely adapted for locomotion and possibly for prey capture. However, key differences are evident on the anterior surface of the distal humerus: *Kostensuchus* gen. nov. has a deep rounded fossa between the supracondylar crests and a well-developed latero-distal protuberance, features absent in baurusuchids. Furthermore, distal to this fossa, *Kostensuchus* gen. nov. bears a distinct step and a proximally facing shelf that extends across the humerus, a character shared with Paleogene sebecids [40] but absent in baurusuchids. The unique morphology of the humerus in *Kostensuchus* gen. nov. suggests potentially greater flexibility and a wider range of movement compared to the more robust and heavily built humerus of baurusuchids.

The ilium of *Kostensuchus* gen. nov. differs in many aspects from that of baurusuchids , in which the acetabulum is very deep and covered by a dorsoventrally thick, horizontally oriented acetabular roof. In contrast, the ilium of *Kostensuchus* gen. nov. has a shallower acetabulum, and the ventral surface of its roof faces ventrolaterally rather than ventrally, resembling the condition of uruguaysuchids [39]. Another difference in the ilium lies in the location of the insertion scars for the *M. iliotibialis*, responsible for hindlimb extension during the step cycle: In baurusuchids, these insertion areas are located at the external end of the laterally projected acetabular roof, while in *Kostensuchus* gen. nov. the insertion areas extend more posteriorly and dorsally and less laterally. The marked differences in the iliac morphology suggest locomotory and/or postural differences in the hip joint between these groups. In

Kostensuchus gen. nov. the femur may have been positioned in a less erect or parasagittal orientation, with a more oblique line of action for the extensor muscles, compared to a more upright posture in baurusuchids.

These anatomical differences, particularly in the forelimb and pelvis, suggest that while both *Kostensuchus* gen. nov. and baurusuchids were large-bodied predators, their locomotor functions and ecological adaptations might have differed. This aligns with the contrasting lifestyles inferred for baurusuchids (terrestrial) and *Peirosaurus* [semiaquatic; 58, 64]. Additional differences between *Kostensuchus* gen. nov. and baurusuchids, are also found in various anatomical regions, including the skull, dentition, axial skeleton, and dermal armor. These further emphasize the morphological and functional disparity of South American notosuchians, possibly due to their distinct phylogenetic histories, varied lifestyles, and feeding strategies, ultimately revealing the ecological diversity of large predatory crocodyliforms from the latest Cretaceous of South America.

Previous research has established that the plesiomorphic limb posture for crocodyliforms was parasagittal, a condition usually associated with terrestriality [65, 66, 67, 68, 69]. Within this framework, aquatic and semiaquatic habits are considered derived features that evolved along the lineage leading to extant crocodiles [65, 68, 70, 71]. Notosuchians have generally been regarded as parasagittal and terrestrial forms [63, 68, 72, 73, 74, 75, 76, 77], retaining this limb posture and associated habits from their basal crocodyliform ancestors [68]. The discovery of the notosuchian *Kostensuchus* gen. nov., with postcranial features indicative of a more sprawling limb posture, together with aquatic adaptations previously described for other peirosaurian lineages (i.e., mahajangasuchids, itasuchids [45, 78, 79, 80, 81], suggests that adaptations to a

more aquatic lifestyle may have evolved more than once within peirosaurians and convergently with eusuchians.

Conclusions

The discovery of *Kostensuchus atrox* gen. et sp. nov. considerably expands the knowledge about the anatomy of broad-snouted peirosaurids, previously known from extremely fragmentary remains from South America and Madagascar. *Kostensuchus* gen. nov. is retrieved as part of a clade of robust, broad-snouted peirosaurids that thrived at the end of the Cretaceous across various regions of Gondwana. The new anatomical information provided by *Kostensuchus* gen. nov. sheds light on both, the similarities and differences between broad-snouted peirosaurids and baurusuchids, the other crocodyliform clade that independently evolved into apex predators during the Cretaceous of Gondwana.

Kostensuchus gen. nov. formed part of the latest Cretaceous ecosystem of southern Patagonia, in a freshwater ecosystem under a temperate to warm climate with seasonal humidity, alongside a diverse fauna of dinosaurs, mammals, and other vertebrates. The broad and high snout of *Kostensuchus* gen. nov., with notably large and robust ziphodont teeth, along with a broad adductor chamber in the skull and deep mandibular ramus, suggests that the new species had powerful bite forces capable of subduing large prey. These features imply that *Kostensuchus* gen. nov. played the role of a top predator within this end-Cretaceous ecosystem.

Supporting information

S1 File. Supplemental figures and text with phylogenetic details.

(PDF)

S2 File. Phylogenetic matrix used in the study in TNT format.

(TNT)

Acknowledgements

Authors would like to thank other members of the crew, including C. Sakata, C. Miyamae, H. Kamei, F. Brissón-Egli, A. Moreno, S. Miner, G. Muñoz, J. De Pasqua, C. Thompson, D. Piazza, G. Lo Coco, A. Misantone, and G. Stoll. A special thanks to Dr. Y. Harashi, General Director of the National Museum of Nature and Science, Japan. Special thanks to Santiago Miner for assistance in scanning and threedimensional modeling of the fossils reported here. Thanks also to Federico Braun for allowing access to his property and Facundo Echeverría and his wife Daphne Fraser (Estancia La Anita) for their valuable help. We thank the Secretaría de Cultura for providing the permits to conduct our projects and explorations in Santa Cruz Province. This research is part of the Argentine-Japanese collaboration, and we thank funding provided by National Geographic Grant NGS 9282-R-22: The End of the Dinosaur Era in Patagonia, Fundação Carlos Chagas Filho de Amparo à Pesquisa do Estado do Rio de Janeiro (Faperj E-26/200.998/2024), Conselho Nacional de Desenvolvimento Científico e Tecnológico (CNPq 303596/2016-3).

Author contributions

Conceptualization: F.E.N., D.P., T.T, M.M. and I.S.C.

Data Collecting: M.M., F.L.A., S.R., G.L.L., S.R., T.T., F.E.N and M.P.I.

Specimen preparation and curation: M.P.I.

Phylogenetic analysis: D.P.

Writing original draft: F.E.N. and D.P.

Writing review and editing: All authors.

References

1. Leanza HA, Apesteguıa S, Novas FE, de la Fuente MS. Cretaceous terrestrial beds from the Neuquén Basin (Argentina) and their tetrapod assemblages. Cretac. Res. 2004; 25(1): 61-87.
2. Novas FE. The Age of Dinosaurs in South America. Indiana University Press, Bloomington, Indiana, 2009.
3. Gasparini Z, Sterli J, Parras A, O'Gorman JP, Salgado L, Varela J, Pol D. Late Cretaceous reptilian biota of the La Colonia Formation, central Patagonia, Argentina: Occurrences, preservation and paleoenvironments. Cretac. Res. 2015; 54: 154-168.
4. Pol D, Baiano MA, Černý D, Novas FE, Cerda IA, Pittman M. A new abelisaurid dinosaur from the end Cretaceous of Patagonia and evolutionary rates among the Ceratosauria. Cladistics. 2024; 40(3): 307-356
5. Novas F, Agnolin Fl, Rozadilla S, Aranciaga-Rolando AM, Brisson-Egli F, Motta MJ, et al. Paleontological discoveries in the Chorrillo Formation (upper

Campanian-lower Maastrichtian, Upper Cretaceous), Santa Cruz Province, Patagonia, Argentina. Rev. Mus. Argent. Cienc. Nat., n.s. 2019; 21(2): 217-293.

6. Agnolin FL, Alvarez-Herrera GP, Aranciaga-Rolando AM, Motta M, Rozadilla S, Verdiquio L, et al. Fossil vertebrates from the Cerro Fortaleza Formation (Upper Cretaceous), Santa Cruz Province, Argentina. Cretac. Res. 2024; 154: 105735.
7. Alarcón-Muñoz J, Vargas AO, Püschel HP, Soto-Acuña S, Manríquez L, Leppe M, et al. Relict duck-billed dinosaurs survived into the last age of the dinosaurs in subantarctic Chile. Sci. Adv. 2023; 9(24): eadg2456.
8. Martinelli A, Forasiepi A. Late Cretaceous vertebrates from Bajo de Santa Rosa (Allen Formation), Río Negro province, Argentina, with the description of a new sauropod dinosaur (Titanosauridae). Rev. Mus. Argent. Cienc. Nat., n.s. 2004; 6(2): 257-305.
9. Salgado L, Coria RA, Ribeiro CMM, Garrido A, Rogers R, Simón ME, et al. Upper Cretaceous dinosaur nesting sites of Río Negro (Salitral Ojo de Agua and Salinas de Trapalcó-Salitral de Santa Rosa), northern Patagonia, Argentina. Cretaceous Research. 2007; 28(3): 392-404.
10. Aranciaga-Rolando AM, Marsà JAG, Agnolin FL, Motta MJ, Rozadilla S, Novas F E. The sauropod record of Salitral Ojo del Agua: An Upper Cretaceous (Allen Formation) fossiliferous locality from northern Patagonia, Argentina. Cretac. Res. 2022a; 129: 105029.
11. Connelly BE, Cardozo MS, Montgomery JD, Rougier GW. New mammals from the Upper Cretaceous Allen Formation (Patagonia, Argentina) and reassessment of meridiolestidan diversity. Cretac Res. 2024; 105935.

12. Moyano-Paz D, Rozadilla S, Agnolin F, Vera E, Coronel MD, Varela AN, et al. The uppermost Cretaceous continental deposits at the southern end of Patagonia, the Chorrillo Formation case study (Austral-Magallanes Basin): Sedimentology, fossil content and regional implications. Cretac. Res. 2022; 130: 105059.
13. Soto-Acuña S, Vargas AO, Kaluza J, Leppe MA, Botelho JF, Palma-Liberona J, et al. Bizarre tail weaponry in a transitional ankylosaur from subantarctic Chile. Nature. 2021; 600(7888): 259-263.
14. Loinaze VS, Vera EI, Moyano-Paz D, Coronel MD, Manabe M, Tsuihiji T, Novas FE. Maastrichtian palynological assemblages from the Chorrillo Formation, Patagonia, Argentina. Rev. Palaeobot. Palynol. 2023; 314: 104893.
15. Vera EI, Perez-Loinaze VS, Moyano-Paz D, Coronel MD, Manabe M, Tsuihiji T, Novas FE. Paleobotany of the uppermost Cretaceous Chorrillo Formation, Santa Cruz Province, Argentina: insights in a freshwater floral community. Cretac. Res. 2022; 138: 105296.
16. Vera EI, Monferran MD, Massaferro J, Sabater LM, Gallego OF, Perez-Loinaze VS, et al. A Maastrichtian insect assemblage from Patagonia sheds light on arthropod diversity previous to the K/Pg event. Commun. Biol. 2023; 6(1): 1249.
17. Rozadilla S, Agnolín F, Manabe M, Tsuihiji T, Novas FE. Ornithischian remains from the Chorrillo Formation (Upper Cretaceous), southern Patagonia, Argentina, and their implications on ornithischian paleobiogeography in the Southern Hemisphere. Cretac. Res. 2021; 125: 104881.
18. Aranciaga-Rolando AM, Motta MJ, Agnolín FL, Manabe M, Tsuihiji T, Novas FE. A large megaraptoridae (Theropoda: Coelurosauria) from upper Cretaceous (Maastrichtian) of Patagonia, Argentina. Sci. Rep. 2022b: 12(1), 6318.

19. Alvarez-Herrera GP, Agnolin F, Rozadilla S, LoCoco GE, Manabe M, Tsuihiji T, Novas FE. New enantiornithine bird from the uppermost Cretaceous (Maastrichtian) of southern Patagonia, Argentina. Cretac. Res. 2023; 144: 105452.
20. Chimento NR, Agnolin FL, Tsuihiji T, Manabe M, Novas FE. New record of a Mesozoic gondwanatherian mammaliaform from Southern Patagonia. Sci. Nat. 2020; 107: 1-7.
21. Chimento NR, Agnolín FL, Tsuihiji T, Manabe M, Novas FE. New gondwanatherian (Mammaliaformes) remains from the Chorrillo formation (Upper Cretaceous) of southern Patagonia, Argentina. Cretac. Res. 2021; 127: 104947.
22. Chimento NR, Agnolín FL, Manabe M, Tsuihiji T, Rich TH, Vickers-Rich P, Novas FE. First monotreme from the Late Cretaceous of South America. Commun. Biol. 2023; 6(1): 146.
23. Mannion PD, Chiarenza AA, Godoy PL, and Cheah YN. 2019. Spatiotemporal sampling patterns in the 230 million year fossil record of terrestrial crocodylomorphs and their impact on diversity. Palaeontology 62: 615–637.
24. Farlow JO, Hurlburt GR, Elsey RM, Britton AR, Langston W. Femoral dimensions and body size of *Alligator mississippiensis*: estimating the size of extinct mesoeucrocodylians. J. Vert. Paleontol. 2005; 25: 354–369.
25. Fernández-Dumont ML, Pol D, Bona P, Apesteguía S. A new species of *Araripesuchus* with durophagous dentition increases the ecological disparity among uruguaysuchid crocodyliforms. J. Syst. Palaeontol. 2024; 22(1): 2373987.

26. Pol D, Nascimento PM, Carvalho AB, Riccomini C, Pires-Domingues RA, Zaher H. A new notosuchian from the Late Cretaceous of Brazil and the phylogeny of advanced notosuchians. PLoS One. 2014; 9(4): e93105.
27. Leardi JM, Pol D, Novas FE, Suárez-Riglos M. The postcranial anatomy of *Yacarerani boliviensis* and the phylogenetic significance of the notosuchian postcranial skeleton. J. Vert. Paleontol. 2015; 35(6): e995187.
28. Goloboff P, Morales M. TNT version 1.6, with a graphical interface for MacOs and Linux, including new routines in parallel. Cladistics. 2023. DOI 10.1111/cla.12524
29. Pol D, Escapa IH. Unstable taxa in cladistic analysis: identification and the assessment of relevant characters. Cladistics. 2009; 25(5): 515-527.
30. Goloboff PA, Szumik CA. Identifying unstable taxa: efficient implementation of triplet-based measures of stability, and comparison with Phyutility and RogueNaRok. Mol. Phylogenetics Evol. 2015; 88: 93-104.
31. Pol D, Rauhut OW, Lecuona A, Leardi JM, Xu X, Clark JM. A new fossil from the Jurassic of Patagonia reveals the early basicranial evolution and the origins of Crocodyliformes. Biol. Rev. Camb. Philos. Soc. 2013; 88(4): 862-872.
32. Pol D, Goloboff PA. The impact of unstable taxa in coelurosaurian phylogeny and resampling support measures for parsimony analyses. Bull. Am. Mus. Nat. Hist. 2020; 440: 97-115.
33. Carvalho IS, Ribeiro LCB, Avilla LS. *Uberabasuchus terrificus* sp. nov., a new Crocodylomorpha from the Bauru Basin (Upper Cretaceous), Brazil. Gondwana Res. 2004; 7(4): 975-1002.

34. Carvalho IS, Vasconcellos FM, Tavares SAS. *Montealtosuchus arrudacamposi*, a new peirosaurid crocodile (Mesoeucrocodylia) from the Late Cretaceous Adamantina Formation of Brazil. Zootaxa. 2007; 1607(1): 35-46.
35. Porter WR, Sedlmayr JC, Witmer LM. Vascular patterns in the heads of crocodilians: blood vessels and sites of thermal exchange. *J*. Anat. 2016; 229(6): 800-824.
36. Witmer LM, Ridgely RC, Dufeau DL, Semones MC. Using CT to peer into the past: 3D visualization of the brain and ear regions of birds, crocodiles, and nonavian dinosaurs. Anatomical imaging: towards a new morphology. 2008; 67-87.
37. Pol D, Rauhut OW, Lecuona A, Leardi JM, Xu X, Clark JM. A new fossil from the Jurassic of Patagonia reveals the early basicranial evolution and the origins of Crocodyliformes. Biol. Rev. 2013; 88(4): 862-872.
38. Montefeltro FC, Andrade DV, Larsson HC. The evolution of the meatal chamber in crocodyliforms. J. Anat. 2016; 228(5): 838-863.
39. Turner AH. Osteology and phylogeny of a new species of *Araripesuchus* (Crocodyliformes: Mesoeucrocodylia) from the Late Cretaceous of Madagascar. Hist. Biol. 2006; 18(3): 255-369.
40. Pol D, Leardi JM, Lecuona A, Krause M. Postcranial anatomy of *Sebecus icaeorhinus* (Crocodyliformes, Sebecidae) from the Eocene of Patagonia. J. Vert. Paleontol. 2012; 32(2): 328-354.
41. Gasparini Z. 1982. Una nueva familia de cocodrilos zifodontes cretácicos de América del Sur. Actas V Congreso Latinoamericano de Geología, Buenos Aires. 1981 4: 317–329.

42. Pol D, Gasparini Z. Crocodyliformes. In Patagonian Mesozoic Reptiles. Bloomington: Indiana University Press. 2007. pp. 116-142.

43. Leardi JM, Pol D, Montefeltro F, Marinho TS, Ruiz JV, Bravo GG, Pinheiro AEP, et al. Phylogenetic nomenclature of Notosuchia (Crocodylomorpha; Crocodyliformes). Bulletin of Phylogenetic Nomenclature. 2024; 1, 3: 44-82.

44. Pinheiro AEP, Gomes-Costa-Pereira PVL, Vasconcellos FM, Brum AS, Souza LG, Costa FR, et al. New Itasuchidae (Sebecia, Ziphosuchia) remains and the radiation of an elusive Mesoeucrocodylia clade. Hist. Biol. 2023; 35(12): 2280-2305.

45. Ruiz JV, Queiroz MV, Martins KC, Godoy PL, Iori FV, Langer MC, Bronzati M. A new Peirosauridae (Crocodyliformes, Notosuchia) from the Adamantina Formation (Bauru Group, Late Cretaceous), with a revised phylogenetic analysis of Sebecia. Anat. Rec. 2024; 1-24.

46. Pochat-Cottilloux Y, Perrier V, Amiot R, Martin JE. A peirosaurid mandible from the Albian–Cenomanian (Lower Cretaceous) of Algeria and the taxonomic content of *Hamadasuchus* (Crocodylomorpha, Peirosauridae). Pap. Palaeontol. 2023a; 9(2): e1485.

47. Martinelli A, Sertich JJ, Garrido AC, Praderio ÁM. A new peirosaurid from the Upper Cretaceous of Argentina: Implications for specimens referred to *Peirosaurus torminni* Price (Crocodyliformes: Peirosauridae). Cretac. Res. 2012; 37: 191-200.

48. Lio G, Agnolín FL, Juarez-Valieri R, Filippi L, Rosales D. A new peirosaurid (Crocodilyformes) from the Late Cretaceous (Turonian–coniacian) of Patagonia, Argentina. Hist. Biol. 2016; 28(6): 835-841.

49. Lamanna MC, Casal GA, Ibiricu LM, Martínez RD. A new peirosaurid crocodyliform from the Upper Cretaceous Lago Colhué Huapi Formation of Central Patagonia, Argentina. Ann. Carnegie Mus. 2019; 85(3): 193-211.
50. Rasmusson-Simons EL, Buckley GA. New material of "*Trematochampsa*" *oblita* (Crocodyliformes, Trematochampsidae) from the Late Cretaceous of Madagascar. J. Vert. Paleontol. 2009; 29(2): 599-604.
51. Gasparini Z, Chiappe LM, Fernandez M. A new Senonian peirosaurid (Crocodylomorpha) from Argentina and a synopsis of the South American Cretaceous crocodilians. J. Vert. Paleontol. 1991; 11(3): 316-333.
52. Buffetaut E, Taquet P. Un nouveau Crocodilien mésosuchien dans le Campanien de Madagascar, Trematochampsa oblita, n.sp. Bull. Soc. géol. Fr. 1979; 2: 183–188.
53. Godoy PL, Ferreira GS, Montefeltro FC, Vila-Nova BC, Butler RJ, Langer MC. Evidence for heterochrony in the cranial evolution of fossil crocodyliforms. Palaeontology. 2019; 61(4): 543-558.
54. Montefeltro FC, Lautenschlager S, Godoy PL, Ferreira GS, Butler RJ. A unique predator in a unique ecosystem: modelling the apex predator within a Late Cretaceous crocodyliform-dominated fauna from Brazil. J. Anat. 2020; 237(2): 323-333.
55. Aubier P, Jouve S, Schnyder J, Cubo J. Phylogenetic structure of the extinction and biotic factors explaining differential survival of terrestrial notosuchians at the Cretaceous–Palaeogene crisis. Palaeontology. 2023: 66(1): e12638.
56. Riff D, Kellner AWA. Baurusuchid crocodyliforms as theropod mimics: clues from the skull and appendicular morphology of *Stratiotosuchus maxhechti* (Upper Cretaceous of Brazil). Zool. J. Linn. Soc. 2011; 163(suppl_1): S37-S56.

57. Holliday JA, Steppan SJ. Evolution of hypercarnivory: the effect of specialization on morphological and taxonomic diversity. Paleobiology. 2004; 30(1): 108-128.

58. Pochat-Cottilloux Y, Rinder N, Perrichon G, Adrien J, Amiot R, Hua S, et al. The neuroanatomy and pneumaticity of *Hamadasuchus* (Crocodylomorpha, Peirosauridae) from the Cretaceous of Morocco and its paleoecological significance for altirostral forms. J. Anat. 2023; 243(3); 374-393.

59. Raigemborn MS, Lizzoli S, Moyano-Paz D, Varela AN, Poiré DG, Perez-Loinaze V, et al. Paleosols of the Maastrichtian dinosaur-bearing Chorrillo Formation (southern Patagonia, Argentina): Paleoenvironmental and paleoclimate implications. Cretac. Res. 2023; 150: 105587.

60. Ibiricu LM, Casal GA, Martínez RD, Alvarez BN, Poropat SF. New materials and an overview of Cretaceous vertebrates from the Chubut Group of the Golfo San Jorge Basin, central Patagonia, Argentina. J. South Am. Earth Sci. 2019; 98: 102460.

61. Darlim G, Montefeltro FC, Langer MC. 3D skull modelling and description of a new baurusuchid (Crocodyliformes, Mesoeucrocodylia) from the Late Cretaceous (Bauru Basin) of Brazil. J. Anat. 2021a; 239(3): 622–662.

62. Godoy PL, Montefeltro FC, Norell MA, Langer MC. An additional baurusuchid from the Cretaceous of Brazil with evidence of interspecific predation among Crocodyliformes. PLoS One. 2014; 9(5): e97138.

63. Sereno P, Larsson H. Cretaceous crocodyliforms from the Sahara. ZooKeys. 2009; 28: 1-143.

64. Sena MV, Cubo J. Inferring the lifestyles of extinct Crocodyliformes using osteoderm ornamentation. Sci. Nat. 2023; 110(5): 41.

65. Parrish JM. The origin of crocodilian locomotion. Paleobiology. 1987; 13(4): 396-414.

66. Nash D. 1975. The morphology and relationships of a crocodilian, *Orthosuchus stormbergi*, from the Upper Triassic of Lesotho. Annls South Afr Mus. 1975; 67: 227-329.

67. Whetstone KN, Whybrow PJ. A “curosorial” crocodilian from the Triassic of Lesotho (Basutoland), Southern Africa. Occ Pap Mus Nat Hist. 1983; 106: 1-37.

68. Pol D. Postcranial remains of *Notosuchus terrestris* Woodward (Archosauria: Crocodyliformes) from the upper cretaceous of Patagonia, Argentina. Ameghiniana. 2005; 42(1): 21-38.

69. Pochat-Cottilloux Y. A review of the non-semiaquatic adaptations of extinct crocodylomorphs throughout their fossil record. The Anatomical Record. 2024; https://doi.org/10.1002/ar.25586

70. Grigg G, Nowack J, Bicudo JEPW, Bal NC, Woodward HN, Seymour RS. Whole-body endothermy: ancient, homologous and widespread among the ancestors of mammals, birds and crocodylians. Biol. Rev. Camb. Philos. Soc. 2022; 97(2): 766-801.

71. de Araújo Sena MV, Montefeltro FC, Marinho TS, Langer MC, Fachini TS, Pinheiro AEP, et al. The cost of living in Notosuchia (Crocodyliformes, Mesoeucrocodylia). Palaeogeogr. Palaeoclimatol. Palaeoecol. 2023; 632: 111855.

72. Turner AH. Osteology and phylogeny of a new species of *Araripesuchus* (Crocodyliformes: Mesoeucrocodylia) from the Late Cretaceous of Madagascar. Historical Biology. 2006; 18(3): 255-369.

73. Nascimento PM, Zaher H. A new species of *Baurusuchus* (Crocodyliformes, Mesoeucrocodylia) from the Upper Cretaceous of Brazil, with the first complete postcranial skeleton described for the family Baurusuchidae. Papéis avulsos de Zoologia. 2010; 50: 323-361.

74. Godoy PL, Bronzati M, Eltink E, Marsola JC, Cidade GM, Langer MC, Montefeltro FC. Postcranial anatomy of *Pissarrachampsa sera* (Crocodyliformes, Baurusuchidae) from the Late Cretaceous of Brazil: insights on lifestyle and phylogenetic significance. PeerJ. 2016; 4: e2075.

75. Faure-Brac MG, Cubo J. Is there any evidence for the presence of extensive fibrolamellar complex in Notosuchia? New data on *Araripesuchus wegeneri*. Lethaia. 2023; 56(3): 1-6.

76. Bonaparte JF. Los vertebrados fósiles de la Formación Río Colorado, de la ciudad de Neuquén y cercanías, Cretácico superior, Argentina. Rev del Mus Argentino Ciencias Nat. 1991; 4: 17-123.

77. Carvalho IS, Gasparini ZB, Salgado L, Vasconcellos FM, Marinho TS. Climate's role in the distribution of the Cretaceous terrestrial Crocodyliformes throughout Gondwana. Palaeogeogr. Palaeoclimatol. Palaeoecol. 2010; 297(2): 252-62.

78. Buckley GA, Brochu CA. An enigmatic new crocodile from the Upper Cretaceous of Madagascar. Spec. Pap. Palaeontol (London). 1999; 60(60): 149-175.

79. Turner AH, Buckley GA. *Mahajangasuchus insignis* (Crocodyliformes: Mesoeucrocodylia) cranial anatomy and new data on the origin of the eusuchian-style palate. J. Vert. Paleontol. 2008; 28(2): 382-408.

80. Pinheiro AEP, Luiz Gomes Costa Pereira, PV, Vasconcellos FM, Brum AS, Souza LG, Costa FR, et al. New Itasuchidae (Sebecia, Ziphosuchia) remains and

the radiation of an elusive Mesoeucrocodylia clade. Hist. Biol. 2023; 35(12): 2280-2305.

81. Sena MV, Andrade RC, Sayão JM, Oliveira GR. Bone microanatomy of *Pepesuchus deiseae* (Mesoeucrocodylia, Peirosauridae) reveals a mature individual from the Upper Cretaceous of Brazil. Cretaceous Research. 2018; 90: 335-348.
82. Bapst DW. paleotree: an R package for paleontological and phylogenetic analyses of evolution. Methods in Ecology and Evolution. 2012; 3(5): 803-807.

FIGURE LEGENDS

Fig 1. Map of the fossil locality of *Kostensuchus* gen. nov. The map shows the locality in southern Patagonia (Santa Cruz Province, Argentina). Modified from [5].

Fig 2. Skull and jaw of *Kostensuchus atrox* gen. et sp. nov. Photographs in (A) right lateral, (B) dorsal, and (C) ventral views. Interpretative drawings in (D) right lateral, (E) dorsal, and (F) ventral views. Abbreviations: ang, angular; ap, anterior palpebral; de, dentary; ec, ectopterygoid; fr, frontal; j, jugal; la, lacrimal; mx, maxilla; pa, parietal; pal, palatine; pmx, premaxilla; pnf, perinarial fossa; po, postorbital; pp, posterior palpebral; pt, pterygoid; q, quadrate; qj, quadratojugal; na, nasal; rarp, retroarticular process; sang, surangular; sof, suborbital fossa; spl, splenial; sq, squamosal. Scale bar 5cm.

Fig 3. Skull and jaw of *Kostensuchus atrox* gen. et sp. nov. Photographs and interpretative drawings in (A-B) anterior and (C-D) posterior views. Abbreviations: ang, angular; ap, anterior palpebral; art, articular; bs, basisphenoid; de, dentary; fr, frontal; j, jugal; la, lacrimal; mx, maxilla; ot, otoccipital; pa, parietal; pmx, premaxilla; pnf,

perinarial fossa; pp, posterior palpebral; pt, pterygoid; ptp, posterior pterygoid process; q, quadrate; qj, quadratojugal; na, nasal; rarp, retroarticular process; sang, surangular; socc, supraoccipital; sq, squamosal. Scale bar 5cm.

Fig 4. Virtual threedimensional model of anterior region of lower jaw of *Kostensuchus atrox* gen. et sp. nov. Model in (A) dorsal, (B) ventral, (C) anterior, and (D) right lateral views. Abbreviations: de, dentary; spl, spenial; spp, splenial peg. Scale bar 5cm.

Fig 5. Skeletal reconstruction of *Kostensuchus atrox* gen. et sp. nov. Threedimensional model in left lateral view with preserved bone in light brown and missing elements in light grey. Scale bar 5cm.

Fig 6. Cervical vertebrae of *Kostensuchus atrox* gen. et sp. nov. Threedimensional model of cervicals, from the axis to the seventh cervical vertebrae in (A) left lateral, (B) ventral, and (C) left lateroventral views. Abbreviations: cr, cervical rib; hy, hypapophysis; ns, neural spine; ost, dorsal osteoderm; pp, parapophysis; prz, prezygapophysis. Scale bar 5cm.

Fig 7. Dorsal vertebrae of *Kostensuchus atrox* gen. et sp. nov. Threedimensional model in (A) left ventral, (B) left lateral, and (C) dorsal views. Abbreviations: di, diapophysis; hy, hypapophysis; ns, neural spine; ost, dorsal osteoderm; pp, parapophysis; poz, postzygapophysis; prz, prezygapophysis. Scale bar 5cm.

Fig 8. Shoulder girdle of *Kostensuchus atrox* gen. et sp. nov. Threedimensional model in (A) left lateral view. Abbreviations: cf, coracoid foramen; cgf, coracoid glenoid facet; cor, coracoid; sc, scapula; sgf, scapular glenoid facet; ost, osteoderm. Scale bar 5cm.

Fig 9. Humerus of *Kostensuchus atrox* gen. et sp. nov. Threedimensional model in (A), lateral, (B), posterior, (C), medial, and (D), anterior views. Abbreviations: adf, anterior distal fossa; dpc, deltopectoral crest; dsh, distal shelf; pdf, posterior distal fossa; scr, supracondylar crests. Scale bar 5cm.

Fig 10. Ilium of *Kostensuchus atrox* gen. et sp. nov. Threedimensional model in (A) lateral view. Abbreviations: is, ischium; isp, ischial peduncle; it, insertion area of *M. iliotibialis*; pap, preacetabular process; pop, postacetabular process; pp, pubic peduncle; ost, dorsal osteoderm; sac, supracetabular crest. Scale bar 5cm.

Fig 11. Phylogenetic relationships of *Kostensuchus atrox* gen. et sp. nov. Reduced strict consensus tree obtained in the phylogenetic analysis. Thick bars in terminal branches represent the chronostratigraphic uncertainty for each taxon. Phylogenetic tree was calibrated using the R package Paleotree [82].

Figure 1

Click here to access/download;Figure;Fig 1.tif

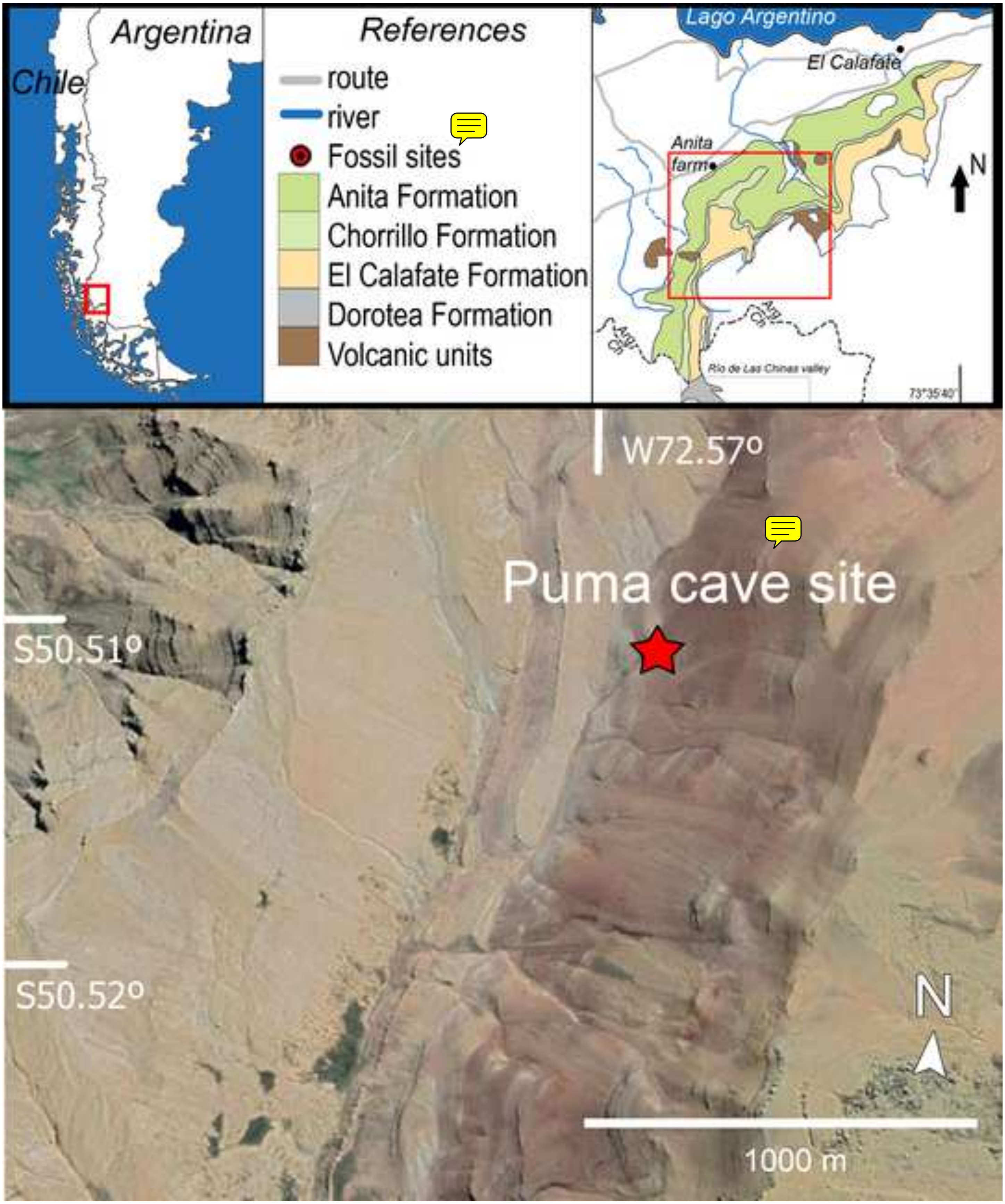

Figure 2

Click here to access/download;Figure;Fig 2.tif

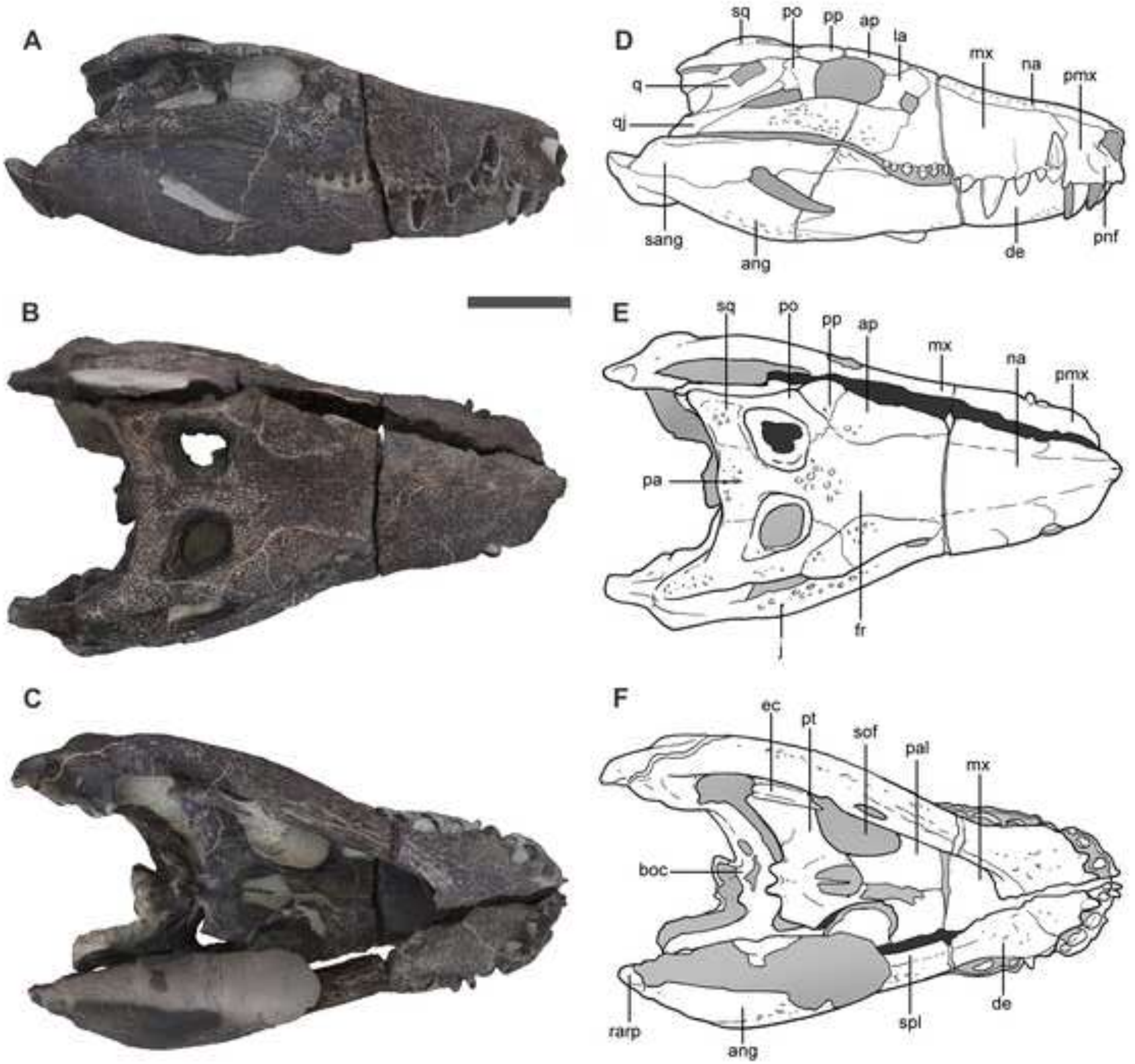

Figure 3

Click here to access/download;Figure;Fig 3.tif

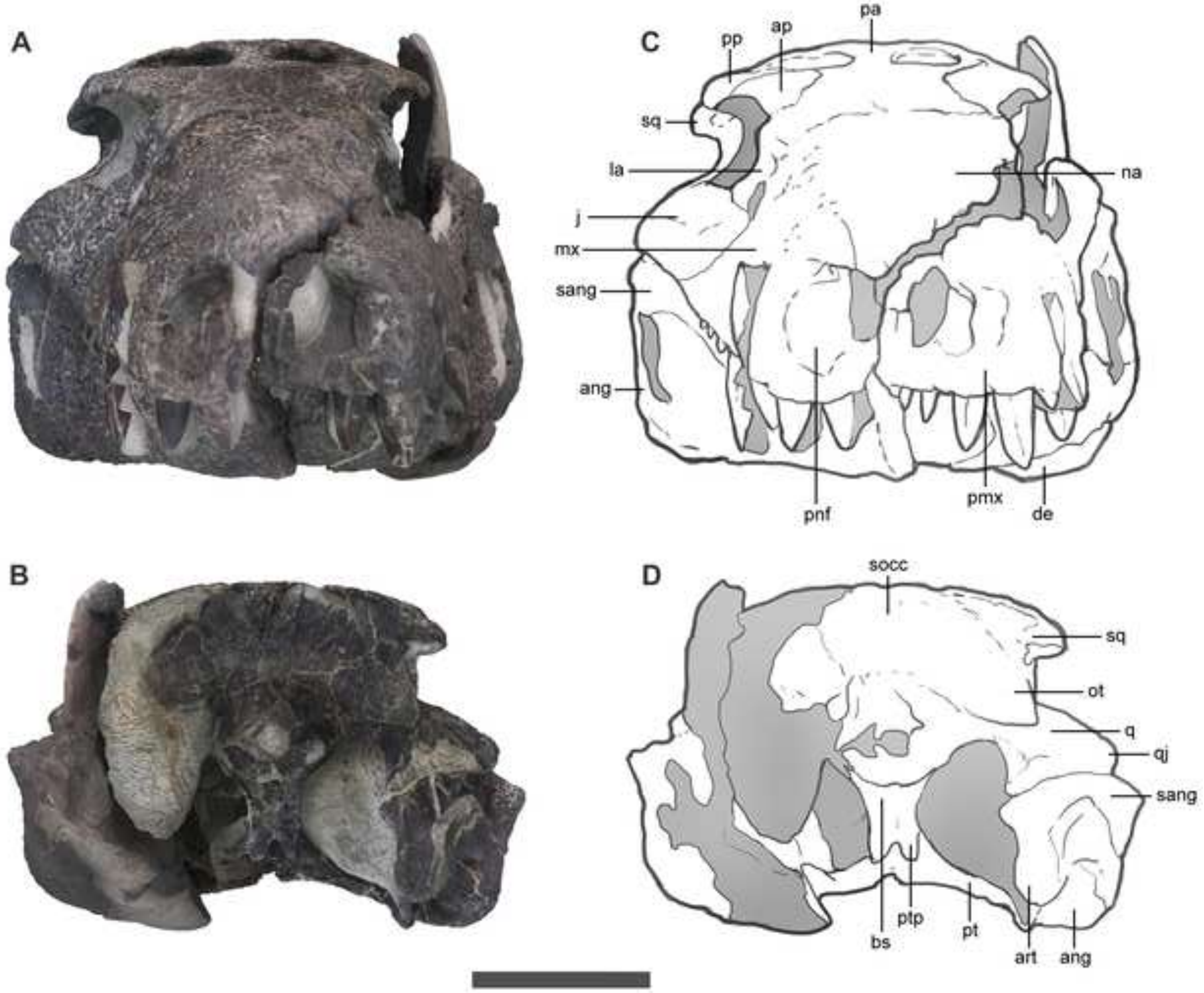

Figure 4

Click here to access/download;Figure;Fig 4.tif

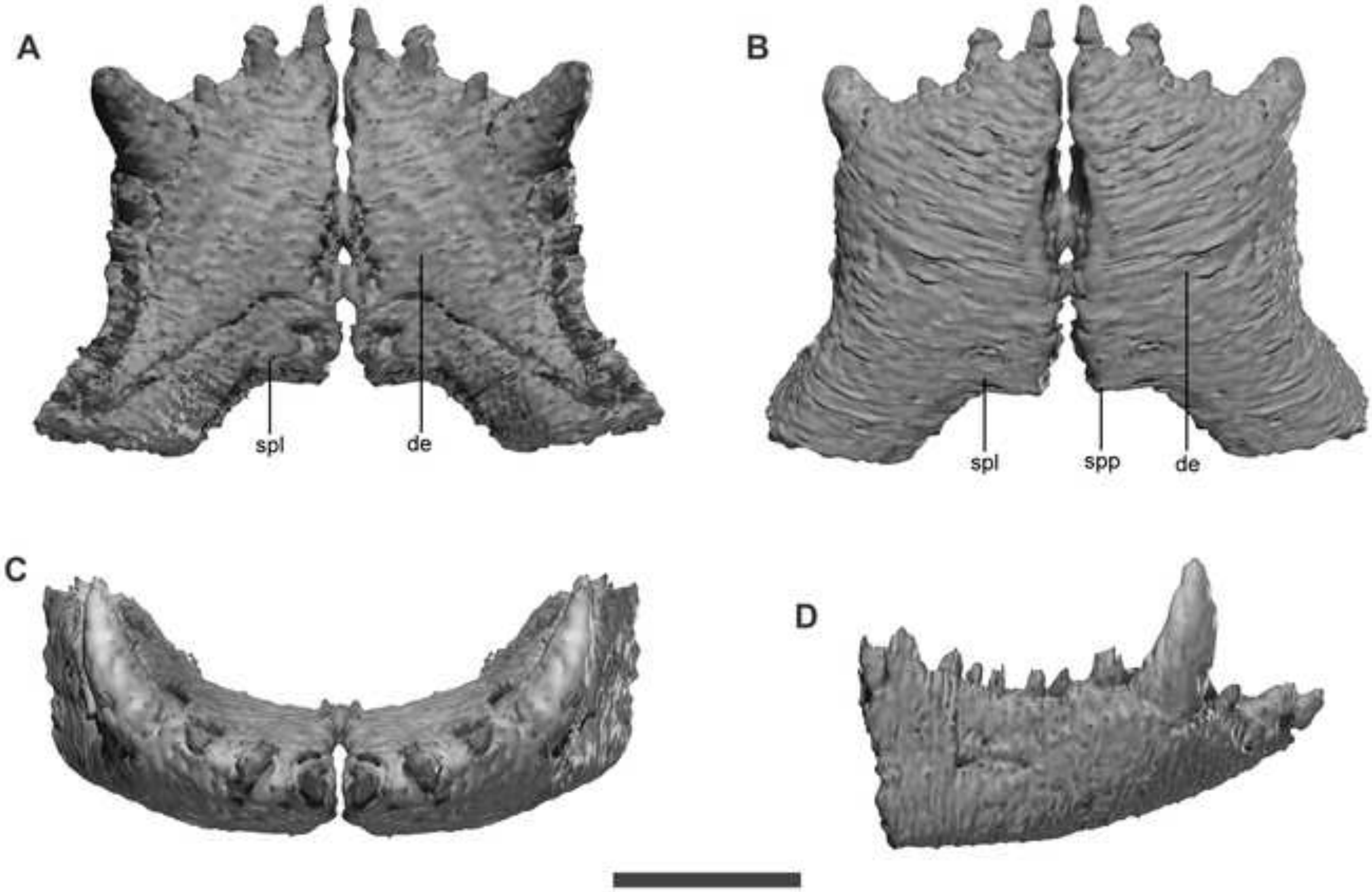

Figure 5

Click here to access/download;Figure;Fig 5.tif

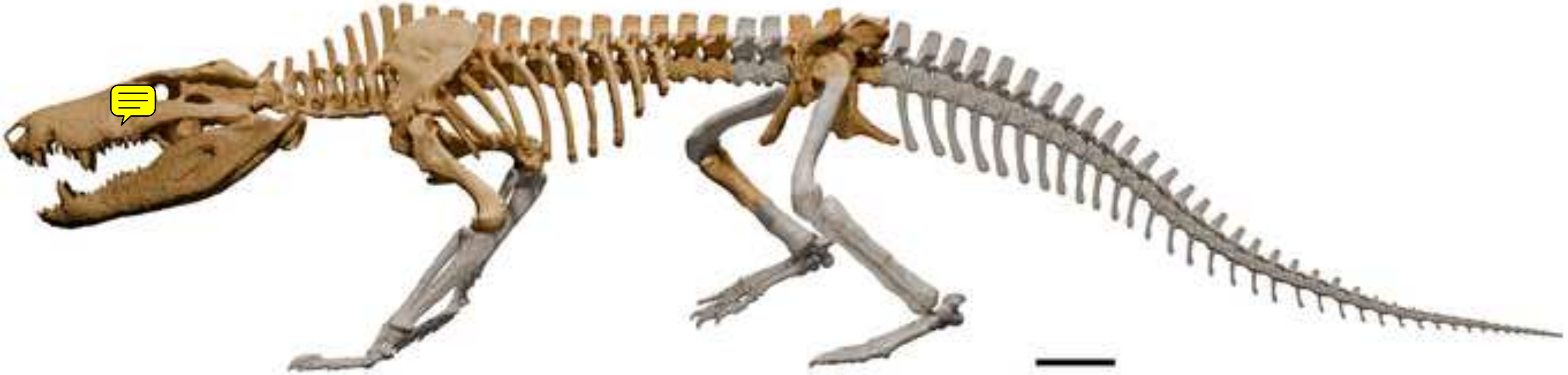

Figure 6

Click here to access/download;Figure;Fig 6.tif

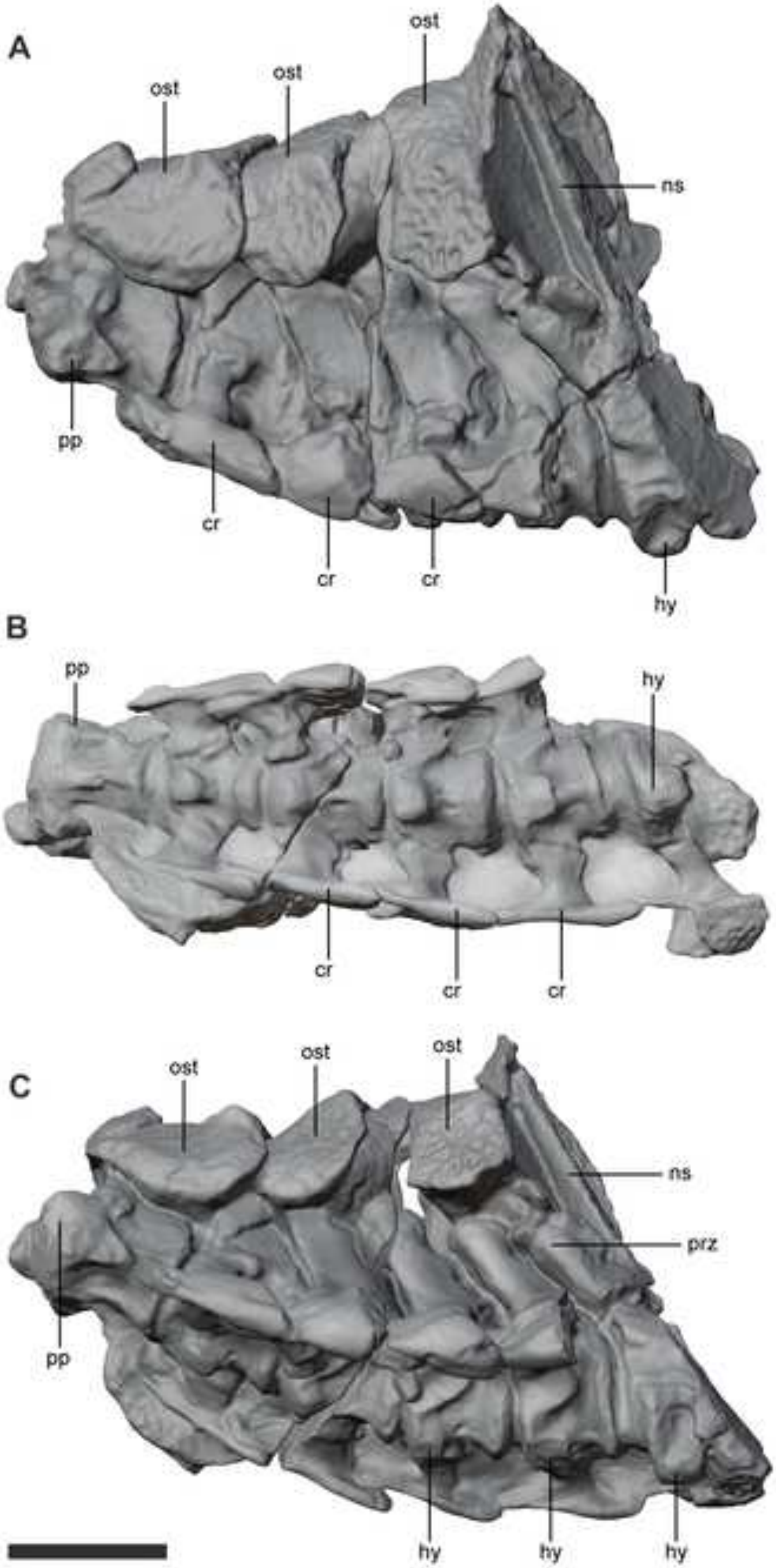

Figure 7

Click here to access/download;Figure;Fig 7.tif

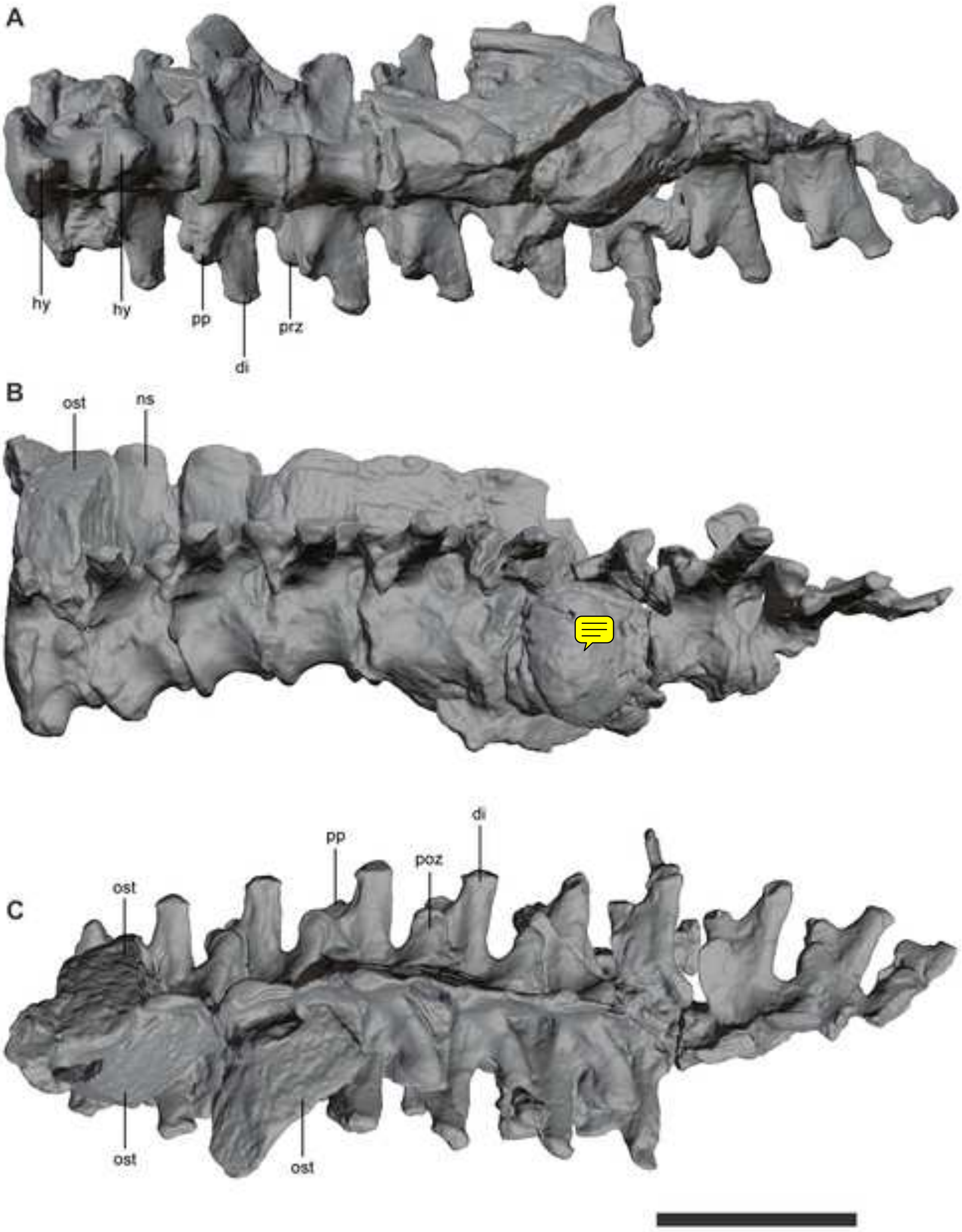

Figure 8

Click here to access/download;Figure;Fig 8.tif

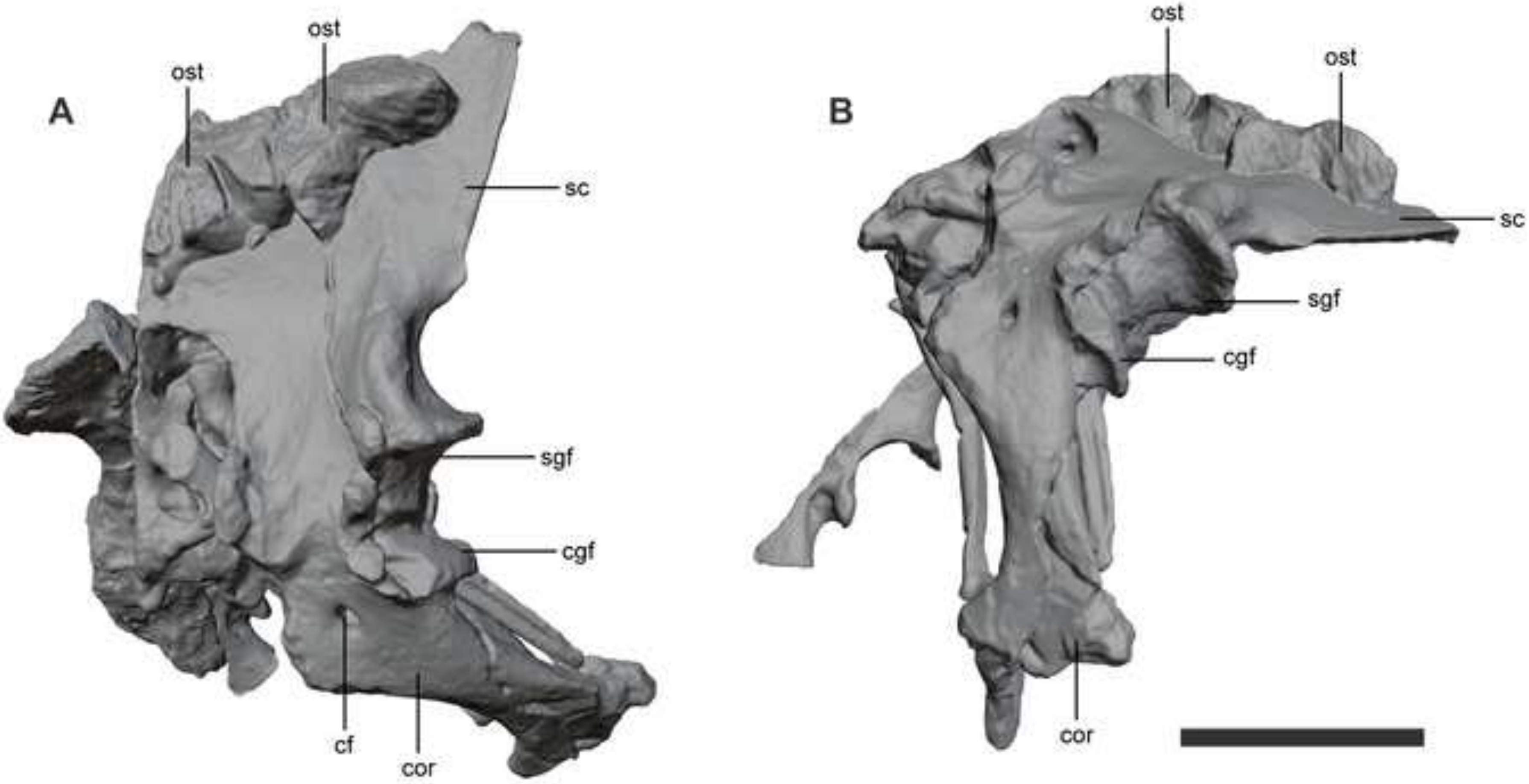

Figure 9

Click here to access/download;Figure;Fig 9.tif

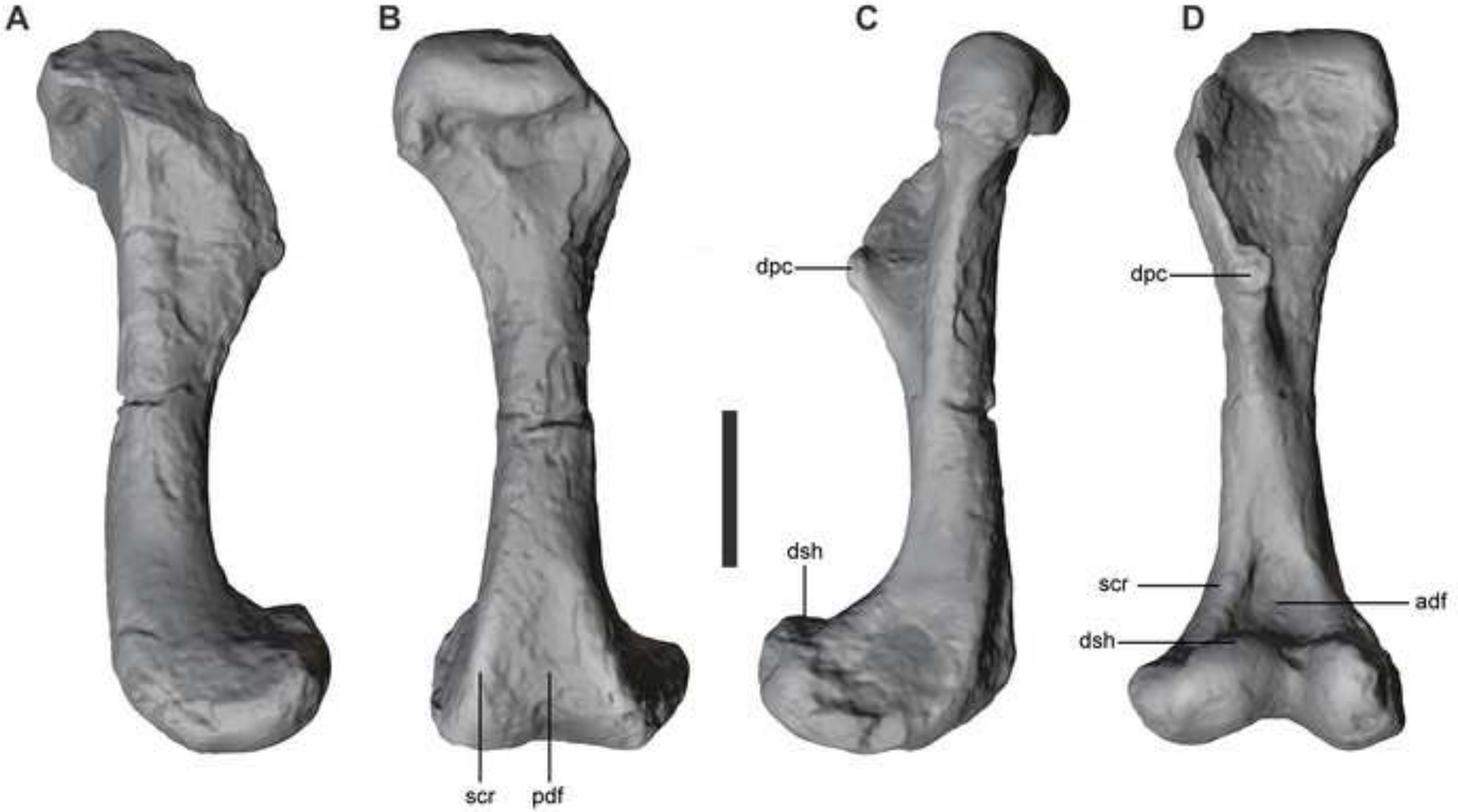

Figure 10

Click here to access/download;Figure;Fig 10.tif

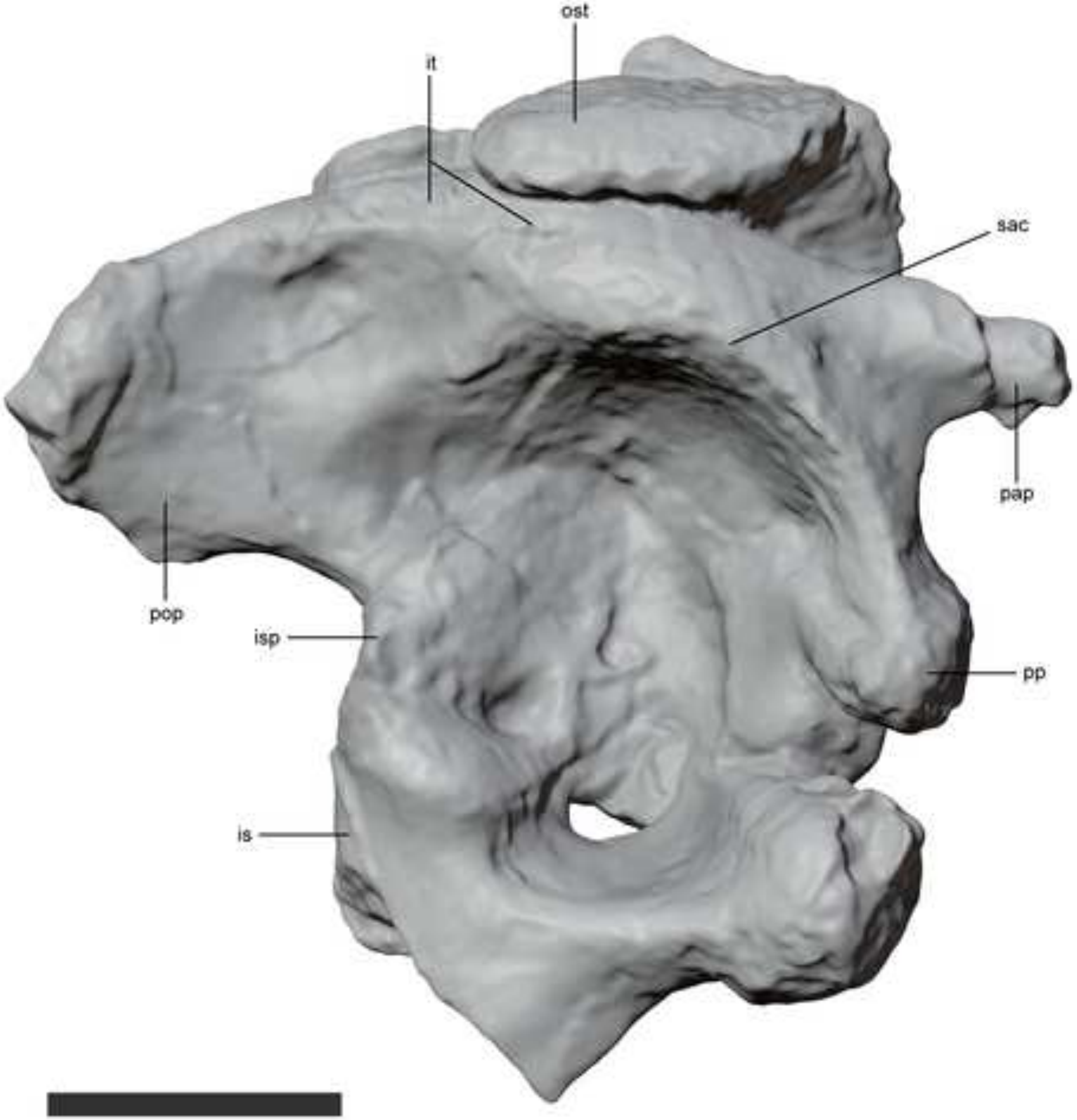

Figure 11

Click here to access/download;Figure;Fig 11-textoutline.eps

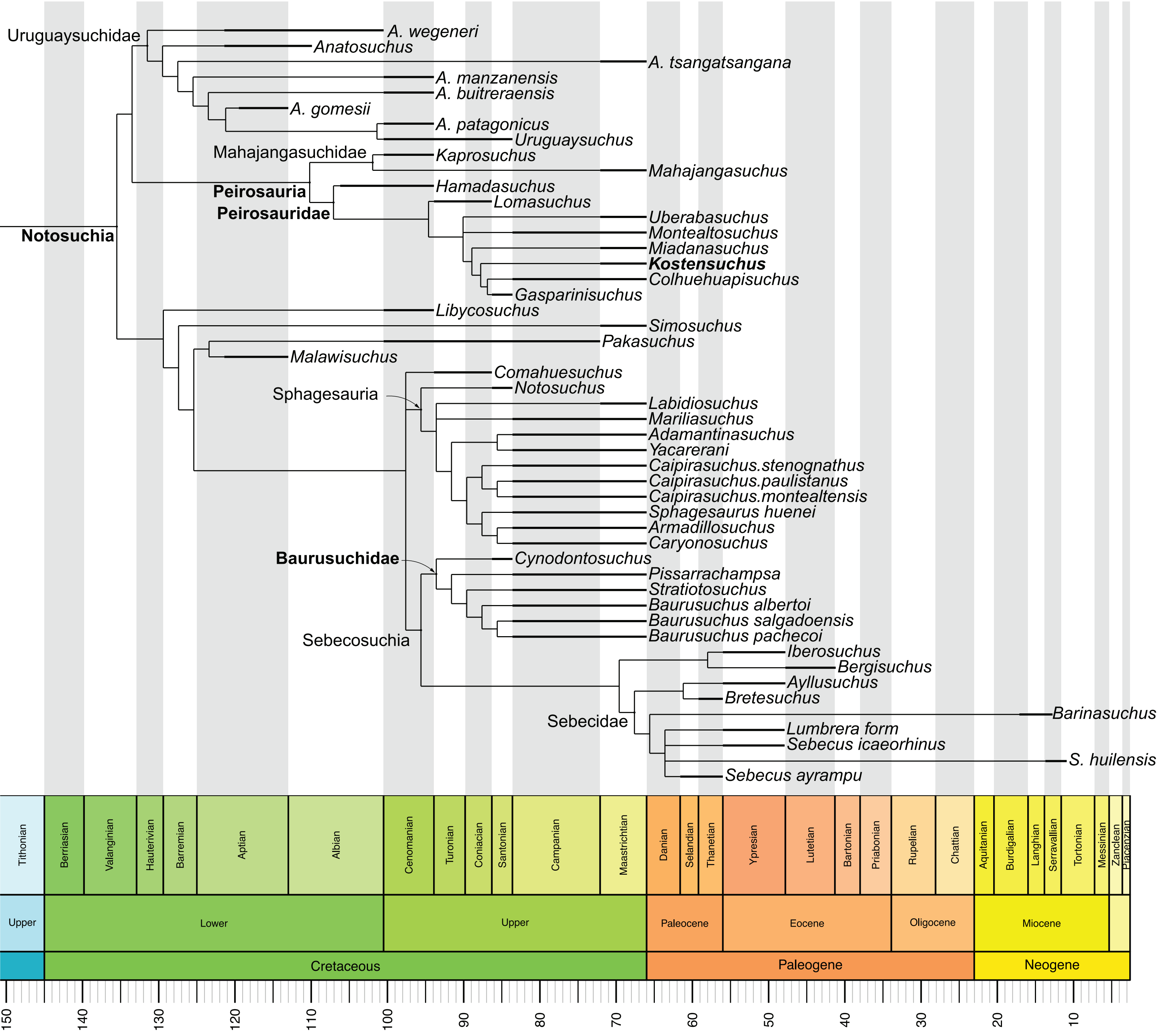

S1 Supplemental figures and phylogenetic details

Click here to access/download
Supporting Information
Novas et al - S1.docx

Click here to access/download
Supporting Information - Compressed/ZIP File Archive
Novas et al - S2 TNT phylogenetic matrix.tnt
