# Supplementary material for: A new large hypercarnivorous crocodyliform from the Maastrichtian of Southern Patagonia, Argentina
Source: PLoS One. 2025 Aug 27;20(8):e0328561. doi: 10.1371/journal.pone.0328561 (PMC12385380; doi:10.1371/journal.pone.0328561)
Supplement: Attachment — Submitted filename: Review - Novas et al. - PONE - peirosaurian from Patagonia.pdf [file pone.0328561.s004.pdf]

A new large hypercarnivorous crocodyliform from the Maastrichtian of Southern Patagonia, Argentina

Fernando E. Novas, Diego Pol, Federico L. Agnolin, Ismar de Souza Carvalho, Makoto Manabe, Takanobu Tsujishi, Sebastián Rozadilla, Gabriel L. Lio & Marcelo P. Isasi

Review

The work is very well written and developed, and the specimen itself is extraordinary for the understanding of Notosuchia, without a doubt.

However, some corrections and modifications are necessary to improve the work.

I believe that future work focused on the taphonomic aspects of the material will be carried out. However, given the interesting fact that it was found in a concretion, it would be appropriate to report part of the process of discovery, collection and preparation of the specimen, even if briefly.

In terms of the morphological description, I felt that more attention should have been paid to the palatal region of the skull, and its elements such as palatines, pterygoid, and ectopterygoids.

As for the postcranium, the osteoderms (dorsal dermal shield) have practically not been described.

In the discussions, when commenting on the differences in the paleofaunas of the southern and northern Patagonia and Brazil, it should be noted that *Barreirosuchus franciscoi* is also a broad-snouted taxon of Peirosauria with large body mass. There is also evidence of megaraptors in the southeastern region of Brazil.

Other comments and suggested changes are highlighted below, in addition to the revised PDF file.

Pg 3

line 10

..."completes the predatorial component"? Finalist phrase! There may be other future discoveries related to predators, even among mammals, for example.

I suggest:

"The new crocodyliform adds to the predatory components of terrestrial ecosystems at high paleolatitudes by the end of the Cretaceous Period."

Pg4

line 3

[e.g., 7, 13].

Pg 5 Methods

It was mentioned that the specimen was preserved through concretions. It would be interesting to report in the Methods how the specimen was collected and prepared.

Pg 8

line 1

From the images, this does not appear to be the case. The craniomandibular articulation appears to be located above the level of the teeth for both the maxilla and the dentary.

line 14

Provide the collection numbers of the specimens to which you refer (compared). After all, one or a few individuals do not represent the average cranial length for a given taxon.

line 23

Uberabasuchus,

Pg 10

line 16

"…in *Uberabasuchus* the snout…" - The bar, not the snout itself.

line 25

Double "and"

Pg 11

line 8

Very interesting feature, but such anatomical characteristic is not seen in the figures of the article, only in the image of the Supplementary Information (Figure S1).

I suggest placing the first two images of the Supplementary Information in the article itself, they are very good.

line 22

Redundant section.

line 25

Requires a figure.

Pg 12

line 14

Suggestion...

"The ascending process of the jugal is superficial at its anterior end, rather than inserted medially as in neosuchians."

line 24

“. *Hamadasuchus*.“ – Loose…

Pg 13

line 3 from bottom to top

Despite the palpebrals, the frontal in *Kostensuchus* appears to form the primary orbital border (see Andrade & Hornung, 2011 - periorbital morphology of *Goniopholis*).

Pg 14

line 11

Confusing sentence. Rephrase.

line 15

Its is clearly broader, but not "much broader condition".

Pg 15

line 5

skull roof

Pg 16

Concise and excellent description of the morphology of the dorsal and lateral regions of the skull. But what about the palatal region, nothing remarkable about the choana, the suborbital fenestra, the pterygoid and ectopterygoids?

line 3 from bottom to top

What is the percentage of symphysis for the *Kostensuchus*?

Pg 17

line 8 from bottom to top

external mandibular fenestra

Pg 19

line 10

Uberabasuchus

line 14

foramen aereum

line 17

If the ridge is continuous with the surangular counterpart, then it is better to standardize the term - transversal ridge.

line 3 from bottom to top

To avoid any confusion with more complex dentitions (e.g., *Simosuchus*), I prefer here the term "teeth with subtriangular or lanceolate crowns", used in Prasad & Broin (2002).

Pg 20

line 2

I prefer the term "carinae".

Pg 21

line 7 from bottom to top

knob-like

Pg 22

line 1

diapophysis

line 8

wing-shaped

Pg 23

line 8

I don't think it surpass, but clearly hits the shaft midpoint.

line 11

a, instead “and”

line 16

"...along the entire width of distal end of the humerus."

Pg 24

line 7

ischiatic or ischial peduncle

line 9

I'm not seeing this in Figure 10.

The process is long but not longer than acetabulum, to me. It may be necessary to delimit the acetabulum.

line 12

I believe the ischium is not preserved (it was not figured). If so, how do you know that?

I noticed the absence of a description, even a brief one, of the osteoderms (dermal dorsal shield) of the taxon.

Pg 25

line 1 from bottom to top

intermandibularis

Pg 26

line 14

From the upper jaw tooth row or from the maxillary series?

line 20

Goniopholididae

Pg 27

line 8

partially complete

line 9

Please indicate, at least in the caption of Fig 5, which other taxa you used as a comparative basis for this type of reconstruction, since many elements of Kostensuchus, including the manus, pes and the entire hind limbs, were not preserved in the holotype.

Pg 28

line 7

An important aspect to consider Baurusuchidae as including hypercanivorous forms is the reduced tooth count. Sometimes found in sebecids, but not in peirosaurids.

Pg 29

line 2

Lago Colhué Huapi Formation (according Lamanna et al., 2019)

line 8

Unless it has been invalidated, there is evidence of Megaraptora gen. et sp. indet. from Ibirá, southeastern Brazil (Maastrichtian of Adamantina Formation (São José do Rio Preto Fm to some) - Bauru Group) See, Méndez, Novas & Iori, 2012.

line 11

southeastern Brazil.

line 12

Barreirosuchus franciscoi, a large broad-snouted peirosaurid / itasuchid, was recovered from the rocks of the Adamantina Formation, southeastern Brazil (see Iori & Garcia, 2012; Fachini, et al. 2024).

lines 14 to **16**

Reevaluate.

Pg 30

line 5

I think *Bretesuchus bonapartei* should be included.

Pg 31

line 10

Wouldn't it be more accurate: "...that surrounds the epiphysis of the humerus." ?

line 11

As far as I know, only *Sebecus icaeorhinus* had its humerus description published, which would be a small sample for such a generalization.

Pg 32

line 1 from bottom to top

Bibliographic citation 80 is the same as 44.

Pg 33

line 8

I would be more cautious here.

line 4 from bottom to top

Finite component analysis, performed by Montefeltro et al. (2020), indicated that the bite force in baurusuchids was weak and that the animals probably had diverse hunting strategies and were perhaps more aggressive to compensate.

Pg 45

80 - Duplicate bibliography. See 44.

Pg 47

Fig 5 - Please indicate which other taxa you used as a comparative basis (modeling) for this type of reconstruction, since many elements of *Kostensuchus*, including the manus, pes and the entire hind limb were not preserved in the holotype.

Pg 48

Fig 9 - humeral distal shelf (following Pol et al., 2012)

Figure 1

Fossil site – in the *References*. Also, convert the black circle to red.

Does the image below correspond to the square with the red border above?

Indicate this in the caption, in addition to the meaning of the red star.

Figure 5

There's something odd about the skull and mandible here, it looks more like *Hamadasuchus* than *Kostensuchus*. The rostrum seems longer, and there's no antorbital fenestra. The mandible looks quite different from that Figure 2 of *Kostensuchus*.

Check this out.

Figure 7, **B**

Could this swollen region in the vertebral centrum be something natural and, therefore, some kind of bone pathology? Or an artifact of the three-dimensionalization process?

===
