# Supplementary material for: A new large hypercarnivorous crocodyliform from the Maastrichtian of Southern Patagonia, Argentina
Source: PLoS One. 2025 Aug 27;20(8):e0328561. doi: 10.1371/journal.pone.0328561 (PMC12385380; doi:10.1371/journal.pone.0328561)
Supplement: Attachment — Submitted filename: Response to Reviewers.docx [file pone.0328561.s007.docx]

Dear Editor,

We thank the reviewers and the editor for their thorough evaluation of our manuscript. We have followed all suggestions except those for which we provide detailed response below in **bold font**:

Reviewer #1:

The work is very well written and developed, and the specimen itself is extraordinary for the understanding of Notosuchia, without a doubt.
However, some corrections and modifications are necessary to improve the work.
I believe that future work focused on the taphonomic aspects of the material will be carried out. However, given the interesting fact that it was found in a concretion, it would be appropriate to report part of the process of discovery, collection and preparation of the specimen, even if briefly.

**Whereas conducting a taphonomic research could be interesting, it lies outside the scope of this work and especially outside our expertise, as we do not have authors capable of conducting a proper taphonomic analysis. However, we follow the advice of the reviewer and we have now included a report on the process of discovery, collection and preparation of the specimen. Given the structure of the manuscript we have decided to include these details in the supplementary information.**

In terms of the morphological description, I felt that more attention should have been paid to the palatal region of the skull, and its elements such as palatines, pterygoid, and ectopterygoids.

**We have expanded the description of the palate, especially of the palatines and pterygoids that are better exposed than the ectopterygoids.**

As for the postcranium, the osteoderms (dorsal dermal shield) have practically not been described.

**We have included a new paragraph description of the osteoderms**

In the discussions, when commenting on the differences in the paleofaunas of the southern and northern Patagonia and Brazil, it should be noted that Barreirosuchus franciscoi is also a broad-snouted taxon of Peirosauria with large body mass.

There is also evidence of megaraptors in the southeastern region of Brazil.

**Yes, good points, we have modified the text to emphasize the differences with northern Patagonia rather than with Brazil. Restricting the comparison to northern Patagonia makes also more sense because there is chronostratigraphic control that correlate the Chorrillo and the Allen formations, wheras the correlation with the Baurú Group of Brazil is less precise.**

My comments and suggested changes are highlighted and are included in the attached files.

**All changes in the marked-up file have been followed.**

Reviewer #2:

I was requested to review the manuscript titled “A new large hypercarnivorous crocodyliform from the Maastrichtian of Southern Patagonia, Argentina” by Novas et al. The manuscript deals with a beautifully preserved new taxon (Kostensuchus) from the latest Cretaceous (Maastrichtian) of Southern Argentina. Besides the good preservation, this new taxon is very large and helps in the knowledge to a clade only known by very partial remains, “broad-snouted” peirosaurids. Furthermore, illustrations of the fossil materials are adequate and contribute to illustrate the specimen.
The manuscript is clear and well-written. I made some comments (typos and some incomplete sentences), but those should be considered as suggestions by the authors as I am not a native English speaker.

**All changes in the marked up file have been followed.**

I think after some minor changes the manuscript could be accepted for publication. I mention, briefly some of these (which are minor):

1) Figures should be cited more often in the description. I added some cites to this, but only in the begging.

**Corrected, we have added more references to figures in the description.**

2) Some cites are missing when comparing the anatomy of Kostensuchus. E.g., “…unlike the condition of most notosuchians”. That should be followed by a cite or at least the mention of some taxa. See the comment (cites) or (e.g., MENTION SOME TAXA) throughout the text.

**Corrected.**

3) Some particular, even unique features, of Kostensuchus can not be seen in the figures (like the grooves on the premaxilla, mandible, or the lack/change of ornamentation near the antorbital fenestra). I don’t know if that is caused by the poor quality of images, if so label them on the figures.

**We have added a new figure (Figure 4) with anatomical details, see below.**

Regarding the unique condition of the lacrimal of Kostensuchus, that could be illustrated in a very simple one-column figure (especially considering it represent an autapomorphy of the new taxon).

**We have added a new figure (Figure 4) with anatomical details that were not well illustrated in the original figures. These include the maxillary groove, the lacrimal bulge, the autapomorphic depressed lateral surface of lacrimal, the lack of lacrimal antorbital fossa, and the extension of the jugal towards the antorbital fenestra.**

4) Add more labels to the figures, in particular to the ones of the posterior view of the skull (some features are described but not marked like the siphonium) and the cervicodorsal block of vertebrae. In the later, if possible add the number of vertebrae (either D1, D2, D3) as it is useful to evaluate some transitions.

**Corrected.**

5) Finally, a small comment on the humeral anatomy of Kostensuchus. The authors infer a more sprawiling limb orientation during the locomotion of Kostensuchus, mostly based on the anatomy of the ilium. However, some of these features were used as criteria to infer a more parasagittal orientation of the limbs in sebecids and baurusuchians (Riff, 2007; Pol et al., 2012). Contrasting with those taxa, Kostensuchus has a much more robust humerus, which is not common un cursorial parasagittal reptiles. Thus, these features could have other putative functions here (i.e., supporting the weight if the anterior part of the body and head?)?

**We have revised the relevant sentence to reflect a more nuanced interpretation. It now reads:**

**“This shared feature has been interpreted as likely related to a parasagittal posture [40], but another possibility is that it reflects the presence of powerful forelimbs associated with prey capture or dismemberment during feeding.”**

**We also added a sentence in the ilium discussion, where we discuss the possible orientation of the hindlimb to acknowledge the limitations of the current data and the need for further evidence:**

**“More remains of the hindlimb, especially of the femur, however, would be needed to test this hypothesis.”**

Figure 9:You mention this distolateral feature. Label it

**We have labeled the anterior distal fossa already.**

Thank you for considering our manuscript. We look forward to the opportunity to share our findings with the scientific community through PLOS ONE. Please do not hesitate to contact me if additional information is required.

Sincerely,

Diego Pol

Museo Argentino de Ciencias Naturales “Bernardino Rivadavia” (MACN)

Buenos Aires, Argentina
